# Clinicopathological characteristics, genetic aberrations, and optimized treatment strategies in double-hit and triple-hit lymphoma: a multi-center cohort study

**DOI:** 10.1186/s43556-025-00346-8

**Published:** 2025-12-09

**Authors:** Yi-Ge Shen, Meng-Meng Ji, Qing Shi, Xiao-Lei Wei, Lei Fan, Ting-Bo Liu, Yao Liu, Li-Hua Dong, Ai-Bin Liang, Liang Huang, Hui Zhou, Hong-Hui Huang, Shen-Miao Yang, Xiao-Bo Wang, Yu-Yang Tian, Zun-Min Zhu, Ou Bai, Fei Li, Wen-Yu Shi, Bin Xu, Xin Wang, Ke-Qian Shi, Wei Tang, Hong-Mei Yi, Si-Yuan Chen, Zhong Zheng, Shu Cheng, Peng-Peng Xu, Wei-Li Zhao, Li Wang

**Affiliations:** 1 State Key Laboratory of Medical Genomics, National Research Center for Translational Medicine at Shanghai, Shanghai Institute of Hematology, Ruijin Hospital Affiliated to Shanghai Jiao Tong University School of Medicine, 197 Rui Jin Er Road, Shanghai, 200025 China; 2 Department of Hematology, Nanfang Hospital, Southern Medical University, Guangzhou, China; 3 Department of Hematology, Jiangsu Province Hospital, The First Affiliated Hospital With Nanjing Medical University, Jiangsu, China; 4 Department of Hematology, Fujian Provincial Key Laboratory On Hematology, Fujian Medical University Union Hospital, Fujian Institute of Hematology, Fuzhou, China; 5 Department of Hematology-Oncology, Chongqing University Cancer Hospital, Chongqing, China; 6 Department of Hematology, The Affiliated Cancer Hospital of Zhengzhou University, Henan Provincial Institute of Hematology, Zhengzhou, China; 7 Department of Hematology, School of Medicine, Tongji Hospital, Tongji University, Shanghai, China; 8 Department of Hematology, Tongji Hospital, Tongji Medical College, Huazhong University of Science and Technology, Wuhan, China; 9 Department of Lymphoma & Hematology, The Affiliated Cancer Hospital of Xiangya School of Medicine, Hunan Cancer Hospital, Central South University, Changsha, China; 10 Department of Hematology, Ren Ji Hospital, Shanghai Jiao Tong University School of Medicine, Shanghai, China; 11 National Clinical Research Center for Hematologic Disease, Peking University Institute of Hematology, Peking University Peoples Hospital, Beijing, China; 12 Department of Hematology, The Second Affiliated Hospital of Dalian Medical University, Dalian, China; 13 Department of Hematology, Hainan Cancer Hospital, Haikou, China; 14 Department of Hematology, Henan Provincial People’s Hospital, Zhengzhou University, Zhengzhou, China; 15 Department of Hematology, The First Hospital of Jilin University, Changchun, China; 16 Center of Hematology, The First Affiliated Hospital of Nanchang University, Nanchang, China; 17 Department of Oncology, Affiliated Hospital of Nantong University, Nantong, China; 18 Department of Hematology, The First Affiliated Hospital of Xiamen University, Xiamen, China; 19 Department of Hematology, Shandong Provincial Hospital, Shandong University, Jinan, Shandong China; 20 Department of Hematology, The First People’s Hospital of Yunnan Province, Kunming, China; 21 Department of Pathology, Ruijin Hospital Affiliated to Shanghai Jiao Tong University School of Medicine, Shanghai, China; 22 Laboratory of Molecular Pathology, Pôle de Recherches Sino-Français en Science du Vivant Et Génomique, Shanghai, China

**Keywords:** Aggressive B-cell lymphoma, Double-hit, Triple-hit, Genetic characteristics, Targeted therapy, Autologous hematopoietic stem cell transplantation

## Abstract

High-grade B-cell lymphoma with MYC and BCL2 and/or BCL6 rearrangements constitutes a distinct clinicopathological entity characterized by aggressive behavior, inherent resistance to conventional immunochemotherapy, and suboptimal clinical outcomes. Within our cohort, MYC/BCL2 rearrangements defined double-hit lymphoma (DHL), MYC/BCL6 as DHL-BCL6, and concurrent MYC/BCL2/BCL6 as triple-hit lymphoma (THL). Here, we delineated the clinical characteristics and genetic aberrations of 112 DHL/THL patients to investigate the factors influencing lymphoma relapse and optimize treatment strategies. Compared to 80 DHL-BCL6 patients, DHL/THL manifested distinct features, including an increased prevalence of the germinal center B-cell-like subtype and co-expression of MYC/BCL2, and demonstrated significant associations with abbreviated progression-free and overall survival. Univariate and multivariate analyses identified Ann Arbor stage and serum lactate dehydrogenase elevation as independent prognostic determinants. Therapeutic intensification employing R-DA-EDOCH was correlated with enhanced survival outcomes, while consolidative autologous stem cell transplantation significantly improved prognosis in patients who achieved remission after first-line immunochemotherapy. Regarding genetic aberrations, oncogenic mutations were detected in 102 evaluable patients. *EZH2* mutation occurred more frequently in DHL/THL, while *TNFRSF14* mutation exhibited greater prevalence in THL. The EZB genotype was predominantly observed in DHL/THL patients, and those with *TP53* abnormalities exhibited a further diminished prognosis. In terms of the immune microenvironment, the depleted lymphoma microenvironment (LME-DP) subtype, characterized by diminished immune cell infiltration, demonstrated a propensity for increased frequency in DHL/THL patients. Collectively, these findings advance the comprehensive understanding of DHL/THL pathobiology, underscoring the imperative for novel targeted agents and therapeutic approaches.

**Supplementary Information:**

The online version contains supplementary material available at 10.1186/s43556-025-00346-8.

## Introduction

Large B-cell lymphoma, the most common type of non-Hodgkin’s lymphoma (NHL), represents heterogeneous pathological, biological, and clinical characteristics [1]. Conventional cytogenetics analysis identifies patients with translocation of MYC and BCL2 and/or BCL6 as a subgroup of aggressive B-cell lymphoma with poor prognosis and phenotypic proximity to Burkitt lymphoma [2]. Owing to their unique features, high-grade B-cell lymphoma with MYC and BCL2 and/or BCL6 rearrangements (known as double-hit or triple-hit lymphoma, DHL/THL) has been defined as a disease category in the World Health Organization (WHO) 2016 classification, accounting for approximately 5–15% of newly diagnosed large B-cell lymphoma [3]. Among them, DHL has cytogenetic characteristics of either MYC and BCL2 gene rearrangement (DHL-BCL2) or MYC and BCL6 gene rearrangement (DHL-BCL6), while THL involves the translocation of all three genes. Recently, DHL-BCL2 has been found to share the same morphological and biologic characteristics as THL, including large, intermediate, or blastoid cells, the germinal center B-cell-like (GCB) subtype [4], with recurrent mutations in *BCL2*, *CREBBP*, *EZH2*, and *KMT2D* [5]. In contrast, DHL-BCL6 exhibits immunoblastic cells, activated B-cell-like (ABC) subtype, and has less cytogenetic complexity as compared to DHL-BCL2 and THL [4]. Therefore, in the WHO 2022 classification [6], DHL-BCL6 is categorized as diffuse large B-cell lymphoma, not otherwise specified (DLBCL-NOS). Per the WHO 2022 guidelines, the term DHL now specifically denotes DHL-BCL2.

The diagnosis and management of DHL/THL present significant challenges. According to the definition, diagnosis of these entities requires the identification of MYC (8q24) and BCL2 (18q21) and/or BCL6 (3q27) translocations using break-apart and dual-fusion probes by fluorescent in situ hybridization (FISH) [7]. Additionally, compared to DHL-BCL6, DHL/THL patients have a higher proportion of MYC protein-positive and *TP53*mutations, which were associated with treatment resistance [8].

Clinically, DHL/THL displays aggressive features, including higher incidences of advanced-stage disease at diagnosis, and poor prognosis to standard immunochemotherapy with rituximab plus cyclophosphamide, doxorubicin, vincristine, and prednisone (R-CHOP) [9]. Despite the development of several treatment strategies including intensified immunochemotherapy, like R-DA-EPOCH (rituximab plus dose-adjusted etoposide, prednisone, vincristine, cyclophosphamide, and doxorubicin), R-CODOX-M/IVAC (rituximab plus cyclophosphamide, vincristine, doxorubicin, high-dose methotrexate/ifosfamide, etoposide, and high-dose cytarabine), and R-Hyper-CVAD (rituximab plus hyperfractionated cyclophosphamide, vincristine, doxorubicin, and dexamethasone), and the potentially curative option of autologous stem cell transplantation (ASCT), no consensus therapeutic standard for DHL/THL patients was established [10]. Considering the adverse clinical outcomes in DHL/THL patients, insights into the molecular mechanisms of the disease are of great importance for exploring novel treatment strategies.

The literature provides scarce information on a comprehensive differential analysis of the clinical manifestations and genetic attributes distinguishing DHL/THL from DHL-BCL6. Therefore, the clinical and molecular differences between DHL/THL and DHL-BCL6 warrant further investigation, aiming to develop optimal therapeutic strategies for this entity.

In the present study, we focused on elucidating the distinct clinical characteristics, survival outcomes, and molecular profiles distinguishing DHL/THL from DHL-BCL6. Moreover, we sought to determine the efficacy of induction immunochemotherapy and front-line ASCT consolidation in the prognosis of DHL/THL patients.

## Results

### Clinical and pathological characteristics of DHL/THL

A total of 192 patients were analyzed, including 71 patients in the DHL group, 41 patients in the THL group, and 80 patients in the DHL-BCL6 group (Fig. S1). The clinical and laboratory characteristics of the patients were summarized in Table 1. Serum lactate dehydrogenase (LDH) levels were significantly increased in THL, as compared to DHL and DHL-BCL6 (34/41 or 82.9% vs 45/71 or 63.4%, *P* = 0.0288; 34/41 or 82.9% vs 45/80 or 56.2%, *P* = 0.0035, respectively). An increased proportion of MYC and BCL2 proteins co-expression (DE) was also observed in THL (THL vs DHL, 37/41 or 90.2% vs 51/71 or 71.8%, *P* = 0.0222; THL vs DHL-BCL6, 37/41 or 90.2% vs 42/80 or 52.5%, *P* < 0.0001). Meanwhile, patients with DHL and THL showed a remarkably higher prevalence of GCB subtype than those with DHL-BCL6 (59/71 or 83.1% vs 38/80 or 47.5%, *P* < 0.0001; 34/41 or 82.9% vs 38/80 or 47.5%, *P* < 0.0001). No statistically significant difference was observed regarding sex, age, International Prognostic Index (IPI) score, Eastern Cooperative Oncology Group (ECOG) performance status, Ann Arbor stage, or extranodal site involvement among the groups. As for treatment strategies, 87 (45.3%) received R-DA-EDOCH (rituximab plus dose-adjusted etoposide, dexamethasone, doxorubicin, cyclophosphamide, and vincristine), 83 (43.2%) received R-CHOP or R-CHOP-like regimen, 15 (7.8%) received R-CHOP combined with novel targeted agents (R-CHOP + X), and 7 (3.7%) received ZR2 (zanubrutinib, rituximab, and lenalidomide) or IR2 (ibrutinib, rituximab, and lenalidomide) regimen. No statistically significant difference in remission rate was observed among the DHL, THL, and DHL-BCL6 groups. Notably, a comparative analysis revealed higher-risk clinicopathological profiles in DHL-BCL6 versus non-DHL controls (*n* = 955, derived from the LymphPlex algorithm cohort), characterized by elevated IPI, advanced stage, extensive extranodal extension, and DE (Table S1). Furthermore, univariate and multivariate analysis among DHL/THL patients confirmed that Ann Arbor stage was an independent prognostic factor for progression-free survival (PFS) in DHL/THL patients (Hazard Ratio (HR): 2.500, 95% Confidence Interval (CI): 1.206–5.181, *P* = 0.0137), while LDH level independently predicted overall survival (OS) (HR: 2.727, 95% CI: 1.100–6.761, *P* = 0.0303, Table 2). Additionally, in the analysis of DHL-BCL6 prognostic factors, both LDH and cell-of-origin (COO) subtype emerged as independent predictors of PFS in DHL-BCL6 patients (Table S2).

**Table 1:** Clinicopathologic features of 192 aggressive B-cell lymphoma patients regarding MYC, BCL2 and BCL6 rearrangements

Characteristic	Overall (*n* = 192) n (%)	DHL-BCL6 (*n* = 80) n (%)	DHL (*n* = 71) n (%)	THL (*n* = 41) n (%)	*P* value^a^	*P* value^b^	*P* value^c^
Age, y							
Median (range)	56 (22–81)	58 (26–81)	56 (29–79)	54 (22–81)			
≤ 60	117 (60.9)	44 (55.0)	46 (64.8)	27 (65.9)	0.2211	0.9093	0.2511
> 60	75 (39.1)	36 (45.0)	25 (35.2)	14 (34.1)			
Gender							
Female	82 (42.7)	33 (41.2)	33 (46.5)	16 (39.0)	0.5179	0.4436	0.8134
Male	110 (57.3)	47 (58.8)	38 (53.5)	25 (61.0)			
IPI risk group							
0–2	106 (55.2)	47 (58.8)	39 (54.9)	20 (48.8)	0.6361	0.5301	0.2964
3–5	86 (44.8)	33 (41.2)	32 (45.1)	21 (51.2)			
ECOG							
< 2	165 (85.9)	70 (87.5)	61 (85.9)	34 (82.9)	0.7744	0.6711	0.4932
≥ 2	27 (14.1)	10 (12.5)	10 (14.1)	7 (17.1)			
Ann Arbor stage							
I-II	59 (30.7)	28 (35.0)	20 (28.2)	11 (26.8)	0.3683	0.8787	0.3627
III-IV	133 (69.3)	52 (65.0)	51 (71.8)	30 (73.2)			
Extranodal sites							
< 2	112 (58.3)	50 (62.5)	39 (54.9)	23 (56.1)	0.3453	0.9047	0.4956
≥ 2	80 (41.7)	30 (37.5)	32 (45.1)	18 (43.9)			
LDH							
≤ normal	68 (35.4)	35 (43.8)	26 (36.6)	7 (17.1)	0.3728	0.0288	0.0035
> normal	124 (64.6)	45 (56.2)	45 (63.4)	34 (82.9)			
Cell of origin (Hans)							
GCB	131 (68.2)	38 (47.5)	59 (83.1)	34 (82.9)	< 0.0001	0.9814	< 0.0001
Non-GCB	61 (31.8)	42 (52.5)	12 (16.9)	7 (17.1)			
BCL2/MYC DE							
With	130 (67.7)	42 (52.5)	51 (71.8)	37 (90.2)	0.0148	0.0222	< 0.0001
Without	62 (32.3)	38 (47.5)	20 (28.2)	4 (9.8)			
First-line therapy							
R-DA-EDOCH	87 (45.3)	37 (46.3)	30 (42.2)	20 (48.8)	0.8090	0.7690	0.8378
R-CHOP/R-CHOP like	83 (43.2)	33 (41.2)	32 (45.1)	18 (43.9)			
R-CHOP + X	15 (7.8)	6 (7.5)	7 (9.9)	2 (4.9)			
ZR2/IR2	7 (3.7)	4 (5.0)	2 (2.8)	1 (2.4)			
Response to first-line therapy							
CR/PR	146 (76.0)	64 (80.0)	54 (76.1)	28 (68.3)	0.5584	0.3714	0.1533
SD/PD	46 (24.0)	16 (20.0)	17 (23.9)	13 (31.7)			

*Abbreviations*: *DHL-BCL6* DLBCL with MYC and BCL6 rearrangements, *DHL* double hit lymphoma, *THL* triple hit lymphoma, *IPI* International Prognostic Index, *ECOG* eastern cooperative oncology group, *LDH* lactate dehydrogenase, *GCB* germinal center B-cell, *DE* MYC/BCL2 protein co-expression, *R-DA-EDOCH* rituximab plus dose-adjusted etoposide, dexamethasone, doxorubicin, cyclophosphamide, and vincristine, *R-CHOP* rituximab with cyclophosphamide, doxorubicin, vincristine, and prednisone, *R-CHOP* + *X* R-CHOP + novel targeted agents, *ZR2* zanubrutinib, rituximab, and lenalidomide, *IR2* ibrutinib, rituximab, and lenalidomide, *CR/PR* complete/partial remission, *SD/PD* stable/progressive disease

^a^*P* value indicated difference between lymphoma with DHL and lymphoma with DHL-BCL6

^b^*P* value indicated difference between lymphoma with DHL and lymphoma with THL

^c^*P* value indicated difference between lymphoma with DHL-BCL6 and lymphoma with THL

**Table 2:** Univariate analysis and multivariate analysis of prognostic factors in DHL/THL patients

Category	Variables	Progression-free survival				Overall survival			
		Univariate		Multivariate		Univariate		Multivariate	
		HR (95% CI)	*P* value	HR (95% CI)	*P* value	HR (95% CI)	*P* value	HR (95% CI)	*P* value
Age, y	≤ 60	1		1		1		1	
	> 60	1.267 (0.735–2.185)	0.3948	1.308 (0.757–2.261)	0.3366	1.617 (0.843–3.101)	0.1485	1.698 (0.878–3.284)	0.1159
Gender	Female	1		-		1		-	
	Male	1.042 (0.606–1.791)	0.8808	-	-	0.815 (0.423–1.571)	0.5416	-	-
IPI risk group	0–2	1		-		1		-	
	3–5	2.076 (1.208–3.567)	0.0082	-	-	2.701 (1.355–5.385)	0.0048	-	-
ECOG	< 2	1		1		1		1	
	≥ 2	1.224 (0.598–2.506)	0.5808	1.004 (0.482–2.090)	0.9914	1.812 (0.825–3.981)	0.1386	1.593 (0.708–3.586)	0.2608
Ann Arbor stage	I-II	1		1		1		1	
	III-IV	2.407 (1.239–4.674)	0.0095	2.500 (1.206–5.181)	0.0137	2.121 (0.966–4.655)	0.0608	1.952 (0.810–4.700)	0.1360
Extranodal sites	< 2	1		1		1		1	
	≥ 2	1.124 (0.661–1.911)	0.6654	0.730 (0.411–1.295)	0.2818	1.071 (0.560–2.045)	0.8363	0.649 (0.320–4.700)	0.2289
Serum LDH	≤ normal	1		1		1		1	
	> normal	1.880 (1.007–3.512)	0.0476	1.595 (0.836–3.044)	0.1563	2.944 (1.224–7.078)	0.0159	2.727 (1.100–6.761)	0.0303
Cell of origin (Hans)	GCB	1		-		1		-	
	Non-GCB	1.275 (0.672–2.420)	0.4567	-	-	1.626 (0.633–4.179)	0.3127	-	-
BCL2/MYC DE	Without	1		-		1		-	
	With	1.034 (0.521–2.055)	0.9229	-	-	1.501 (0.683–3.297)	0.3121	-	-

*Abbreviations*: *DHL* double hit lymphoma, *THL* triple hit lymphoma, *HR* hazard ratio, *CI* confidence interval, *IPI* International Prognostic Index, *ECOG* eastern cooperative oncology group, *LDH* lactate dehydrogenase, *GCB* germinal center B-cell, *DE* MYC/BCL2 protein co-expression

### Intensive chemotherapy improves survival of DHL/THL

**Fig. 1:**
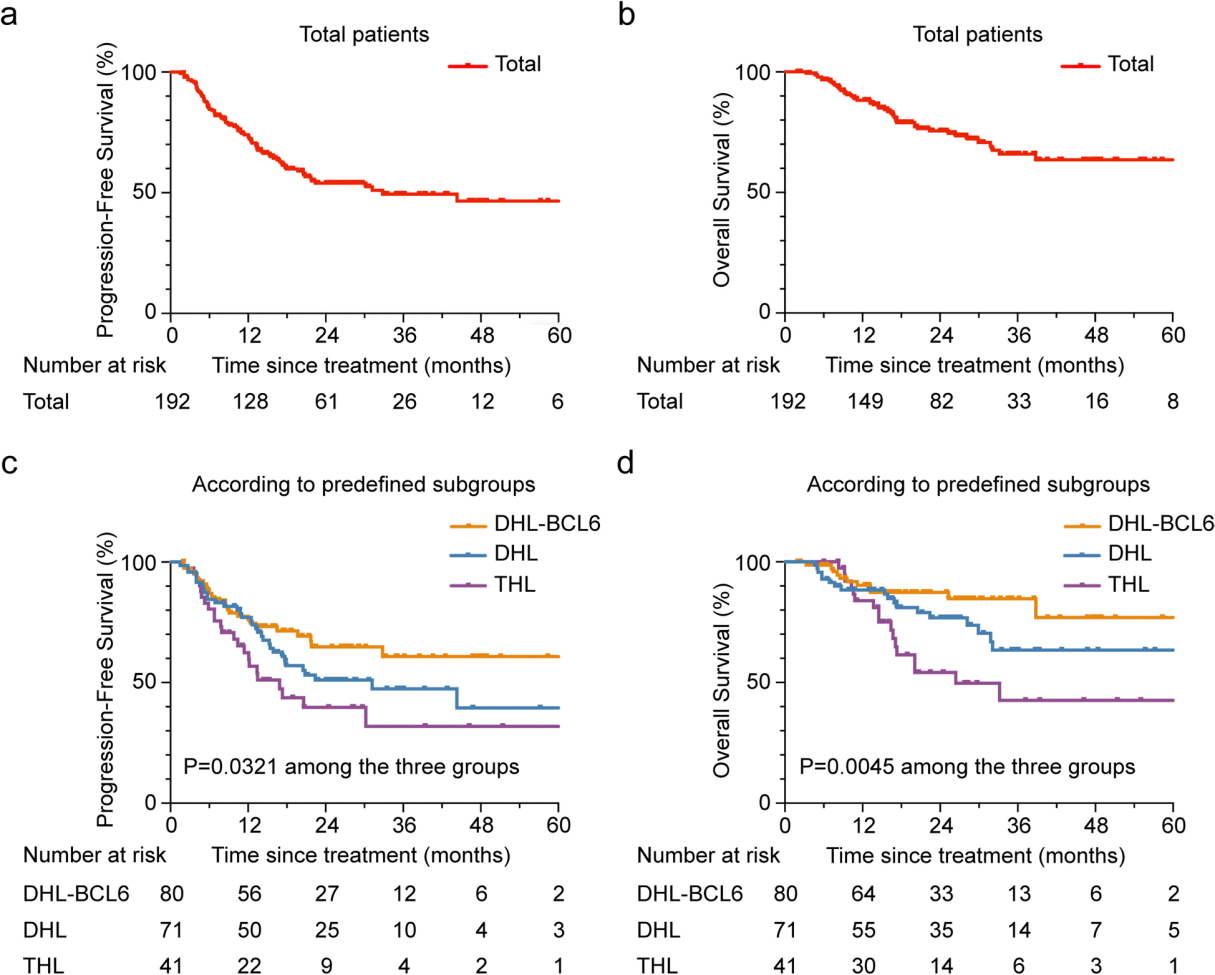
Progression-free survival and overall survival of aggressive B-cell lymphoma patients regarding MYC, BCL2 and BCL6 rearrangements Kaplan–Meier curves of PFS (**a**) and OS (**b**) in total 192 aggressive B-cell lymphoma patients regarding MYC, BCL2 and BCL6 rearrangements. Log-rank test *P* value for intergroup comparisons were shown. Kaplan–Meier curves of PFS (**c**) and OS (**d**) in DHL-BCL6 (*n* = 80), DHL (*n* = 71), and THL (*n* = 41). Log-rank test *P* values among three patient groups was listed. Abbreviations: PFS, progression free survival; OS, overall survival; DHL-BCL6, DLBCL with MYC and BCL6 rearrangements; DHL, double hit lymphoma; THL, triple hit lymphoma

**Fig. 2:**
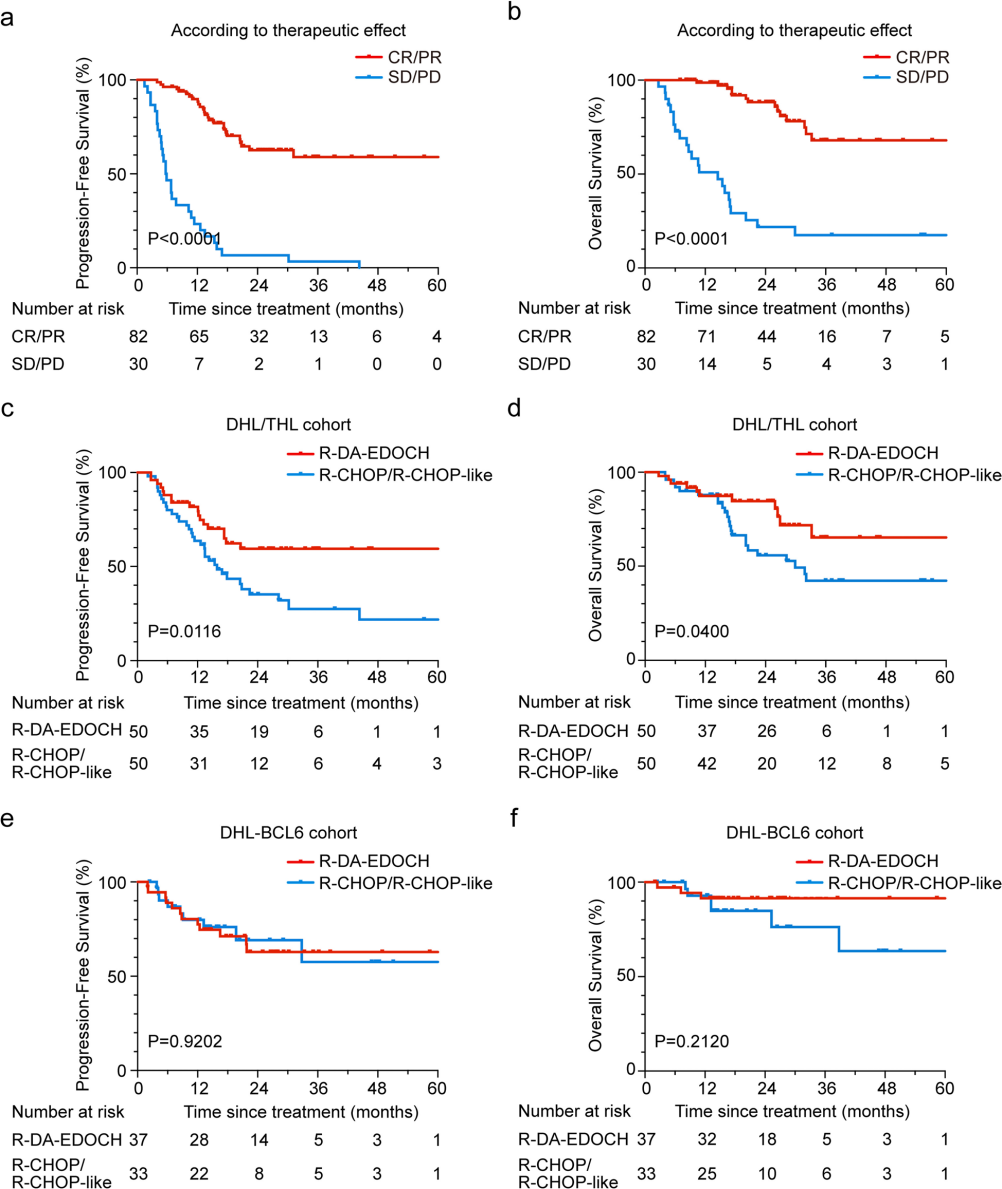
Progression-free survival and overall survival of DHL/THL patients according to clinical treatment Kaplan–Meier curves of PFS (**a**) and OS (**b**) in DHL/THL patients achieving CR/PR (*n* = 82) or SD/PD (*n* = 30) after first-line treatment. Kaplan–Meier curves of PFS (**c**) and OS (**d**) in DHL/THL patients receiving R-DA-EDOCH (*n* = 50), and R-CHOP/R-CHOP-like (*n* = 50) regimen as first-line treatment. Kaplan–Meier curves of PFS (**e**) and OS (**f**) in DHL-BCL6 patients receiving R-DA-EDOCH (*n* = 37) and R-CHOP/R-CHOP-like (*n* = 33) regimen as first-line treatment. Abbreviations: PFS, progression free survival; OS, overall survival; CR/PR, complete/partial remission; SD/PD, stable/progressive disease. DHL-BCL6, DLBCL with MYC and BCL6 rearrangements; DHL, double hit lymphoma; THL, triple hit lymphoma; R-DA-EDOCH, rituximab plus dose-adjusted etoposide, dexamethasone, doxorubicin, cyclophosphamide, and vincristine; R-CHOP, rituximab with cyclophosphamide, doxorubicin, vincristine, and prednisone

With a median follow-up time of 18.8 months (2.0–78.7 months), the 3-year PFS and OS for all patients were 50.5% and 64.4%, respectively (Fig. 1a-b). Patients in the DHL and THL group had dismal survival (3-year PFS: 49.9% and 31.9%; 3-year OS: 61.1% and 40.6%), as compared to those in the DHL-BCL6 group (3-year PFS: 60.8%; 3-year OS: 84.8%, Fig. 1c-d). Significant differences in PFS and OS were observed (*P* = 0.0321 for PFS and 0.0045 for OS). However, compared to those with non-DHL in the LymphPlex algorithm framework, survival outcomes for patients with DHL-BCL6 showed no significant difference (Fig. S2). Among DHL/THL patients achieving complete remission (CR) or partial remission (PR), regardless of the front-line regimen, the 3-year PFS and OS rates were 61.6% and 68.0%, respectively. Survival time was significantly prolonged in the CR/PR patients as compared to the first-line chemo-resistant patients (Fig. 2a-b). Briefly, the 3-year PFS and OS were 59.5% and 65.3% in DHL/THL patients receiving R-DA-EDOCH (*n* = 50), compared to 27.5% and 42.3% in those receiving R-CHOP or R-CHOP-like regimen (*n* = 50, Fig. 2c-d). Patients treated with intensive chemotherapy experienced a significantly improved survival rate compared with those who received R-CHOP or R-CHOP-like (*P* = 0.0116 for PFS and *P* = 0.0400 for OS). Given the limited number of patients receiving alternative regimens, subgroup analyses were not conducted in these treatment groups. PFS and OS according to the treatment regimens in the DHL-BCL6 group were also shown (Fig. 2e-f). No significant differences in outcomes were noted between the different treatments. Relapsed/refractory (R/R) DHL/THL demonstrated a poor prognosis characterized by rapidly progressive disease and potentially fatal outcomes. The cohort exhibited a median PFS of 8.9 months, a median OS of 16.0 months, and a 2-year OS rate of 42.1%. Compared to a contemporaneous cohort of DHL/THL patients in sustained remission, R/R DHL/THL patients exhibited a significantly higher prevalence of high-risk IPI scores (Table S3).

### ASCT is associated with a survival benefit in DHL/THL responders

**Fig. 3:**
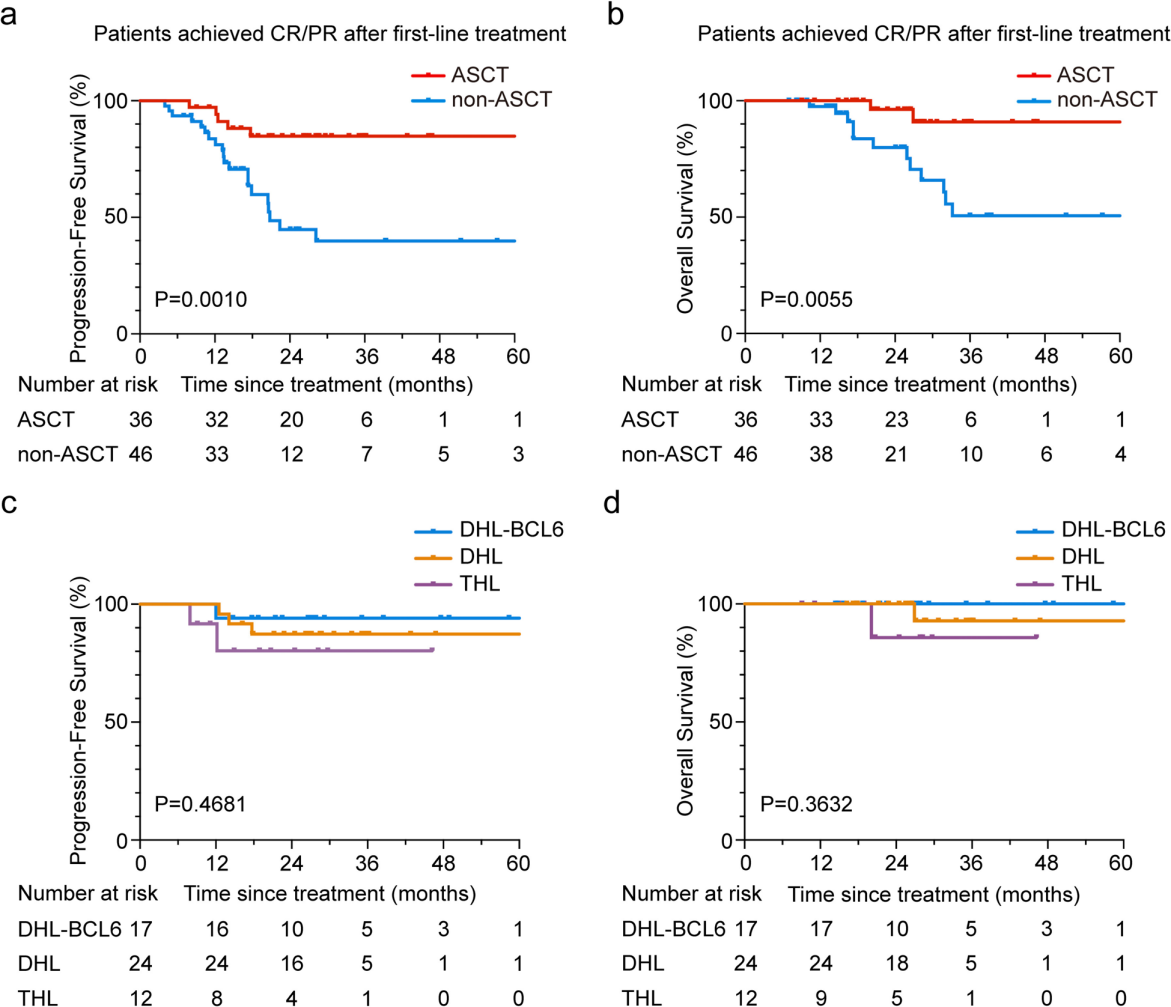
Progression-free survival and overall survival of ASCT and non-ASCT patients after first-line remission Kaplan–Meier curves of PFS (**a**) and OS (**b**) among DHL/THL patients who received ASCT (*n* = 36) and those who did not receive ASCT (*n* = 46) after first-line remission. Kaplan–Meier curves of PFS (**c**) and OS (**d**) among patients who received ASCT in DHL-BCL6 (*n* = 17), DHL (*n* = 24), and THL (*n* = 12) group. Abbreviations: PFS, progression free survival; OS, overall survival; ASCT, autologous stem cell transplantation; DHL-BCL6, DLBCL with MYC and BCL6 rearrangements; DHL, double hit lymphoma; THL, triple hit lymphoma

Among 82 DHL/THL patients achieving CR or PR after first-line treatment, consolidative ASCT was performed in 36 (43.9%). The 3-year PFS and OS rates were 84.8% and 90.9%, respectively, in the ASCT group, while the rates for the non-ASCT group were 39.9% and 50.6%, respectively. Of note, ASCT was associated with improved survival of this selected cohort (*P* = 0.0010 for PFS; *P* = 0.0055 for OS, Fig. 3a-b). When stratified by molecular subtype, ASCT conferred clinically meaningful improvements across DHL, THL, and DHL-BCL6 cohorts, with a more pronounced advantage observed in DHL/THL entities (Fig. 3c-d and Table S4). No significant difference in outcomes was observed between patients achieving CR and PR before ASCT (Fig. S3).

### DHL/THL shares similar genetic features with a high prevalence of *EZH2* mutation

**Fig. 4:**
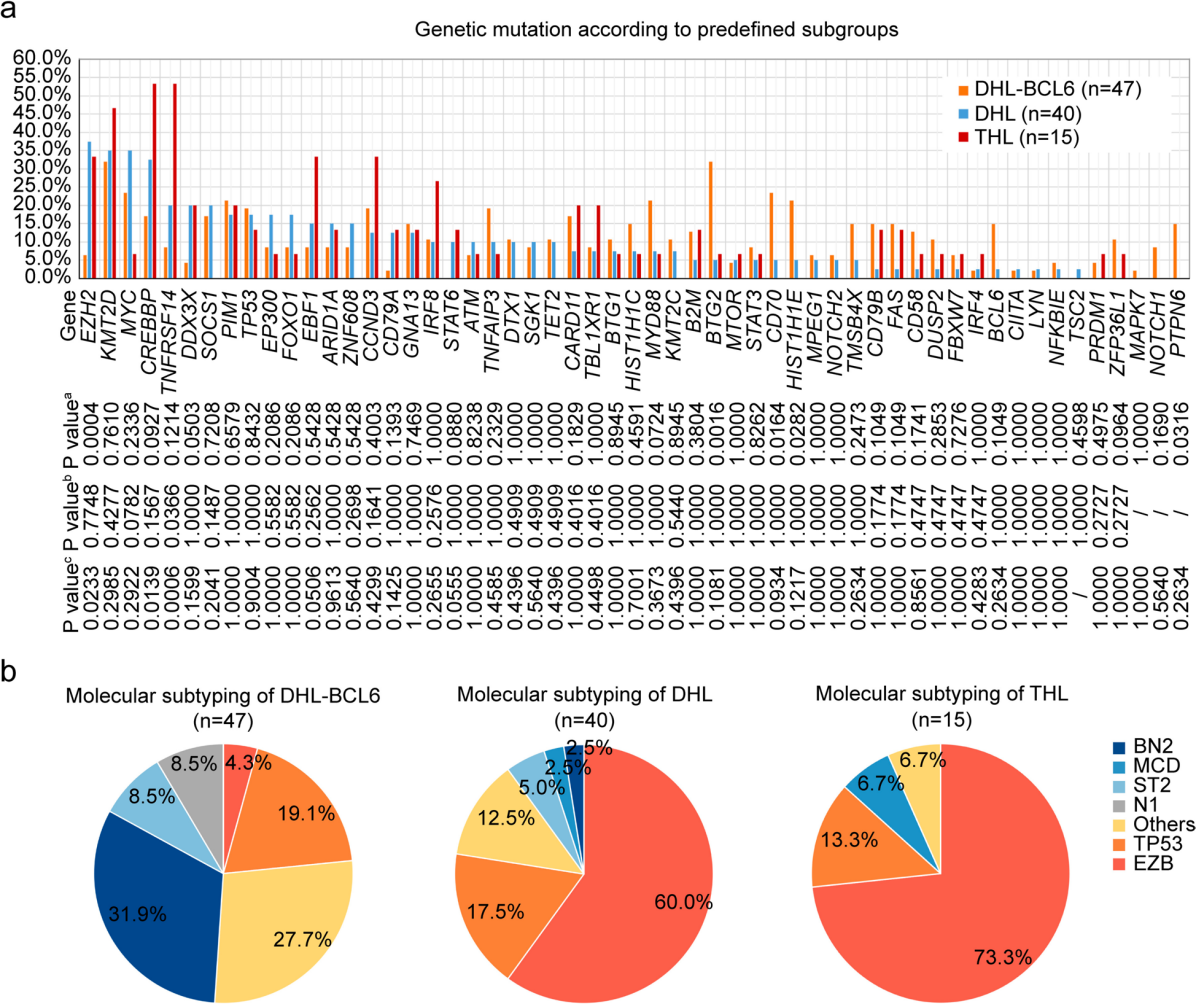
Genetic mutations and molecular subtyping of aggressive B-cell lymphoma patients regarding MYC, BCL2 and BCL6 rearrangements (**a**) Prevalence of genetic mutations in patients with DHL-BCL6 (*n* = 47), DHL (*n* = 40), and THL (*n* = 15). Lower graph indicates *P* values comparing different prevalence in two groups. ^a^*P* value indicated difference between lymphoma with DHL and lymphoma with DHL-BCL6. ^b^*P* value indicated difference between lymphoma with DHL and lymphoma with THL. ^c^*P* value indicated difference between lymphoma with DHL-BCL6 and lymphoma with THL. **b** Prevalence of DLBCL subtypes classified by LymphPlex algorithm in DHL-BCL6 (*n* = 47), DHL (*n* = 40), and THL (*n* = 15). Abbreviations: DHL-BCL6, DLBCL with MYC and BCL6 rearrangements; DHL, double hit lymphoma; THL, triple hit lymphoma

To identify molecular mechanisms associated with poor prognosis, oncogenic mutations were analyzed in 102 (53.1%) of the 192 patients, and pairwise comparisons between different groups were performed (Fig. 4a and Fig. S4). DNA methylation-related *EZH2* gene mutation (37.5% vs 6.4%; *P* = 0.0004) was significantly higher in DHL than in DHL-BCL6. Meanwhile, alterations related to cellular differentiation and immune escape, including *BTG2* (31.9% vs 5.0%; *P* = 0.0016), *CD70* (23.4% vs 5.0%; *P* = 0.0164), *HIST1H1E* (21.3% vs 5.0%; *P* = 0.0282), and *PTPN6* (14.9% vs 0.0%; *P* = 0.0316) gene mutations were more frequently observed in DHL-BCL6 than DHL. The predominant genetic lesions in THL compared to DHL-BCL6 were *TNFRSF14* (53.3% vs 8.5%, *P* = 0.0006), *CREBBP* (53.3% vs 17.0%; *P* = 0.0139), and *EZH2* (33.3% vs 6.4%; *P* = 0.0233). THL and DHL shared similar genetic features, while the increased frequency of *TNFRSF14* gene mutations (53.3% vs 20.0%, *P* = 0.0366) was observed in THL. No notable disparity in the frequency of *TP53* mutations was observed among the three groups. In addition, we compared the genetic alterations between DHL-BCL6 and non-DHL patients, identifying a similarly elevated prevalence of mutations in genes associated with cellular differentiation and immune escape in the DHL-BCL6 subgroup, including *MYC*, *CD70*, *BTG2*, and *IRF8* (Fig. S5a).

According to the LymphPlex algorithm [11], differences in genotyping were shown in Fig. 4b. Among patients with DHL and THL, 60.0% and 73.3% of cases, respectively, were classified into the EZB-like genotype, which is significantly higher than the 4.3% cases in DHL-BCL6 (both *P* < 0.0001). Notably, the BN2-like genotype occurred significantly more frequently in DHL-BCL6 than in DHL (31.9% vs 2.5%; *P* = 0.0004), THL (31.9% vs 0.0%; *P* = 0.0120), and non-DHL patients (31.9% vs 11.1%; *P* < 0.0001; Fig. S5b).

### *TP53* mutation is accumulated in R/R DHL/THL

**Fig. 5:**
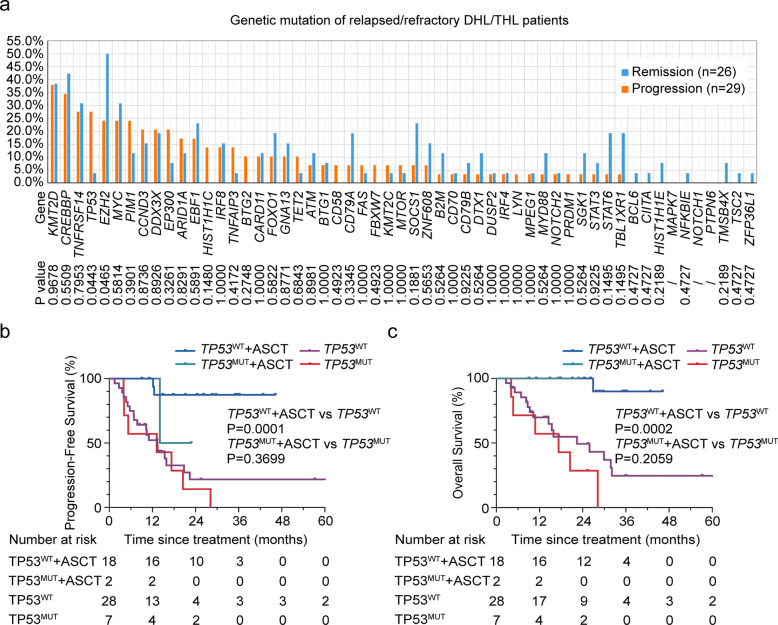
Genetic mutations associated with progressive disease and their impact on survival outcomes in DHL/THL patients (**a**) Prevalence of genetic mutations in DHL/THL patients with sustained remission (*n* = 26) and disease progression (*n* = 29). Lower panel shows *P* values for differences in mutation prevalence between outcome groups. Kaplan–Meier curves of PFS (**b**) and OS (**c**) in DHL/THL patients received ASCT with mutant (*n* = 2) and wild-type *TP53* (*n* = 18), and patients received non-ASCT with mutant (*n* = 7) and wild-type *TP53* (*n* = 28). Log-rank test *P* value for intergroup comparisons were shown. Abbreviations: DHL, double hit lymphoma; THL, triple hit lymphoma; PFS, progression free survival; OS, overall survival; ASCT autologous stem cell transplantation

The genetic mutations in the R/R DHL/THL patients were also described. Patients in the R/R DHL/THL cohort (*n* = 29) presented with a significantly elevated *TP53* mutation frequency (27.6% or 8/29 vs. 3.8% or 1/26, *P* = 0.0443) (Fig. 5a). Although this patient cohort generally showed dismal clinical outcomes, *TP53*^MUT^ patients undergoing ASCT displayed a clinically relevant survival advantage. The lack of statistical significance in both PFS (*P* = 0.370) and OS (*P* = 0.206) should be interpreted in the context of the subgroup's limited sample size (Fig. 5b-c). Meanwhile, significantly superior survival outcomes were observed in *TP53*^WT^ patients undergoing ASCT compared to those without (*P* = 0.0001 for PFS and *P* = 0.0002 for OS). In addition, four elderly R/R patients received chidamide and venetoclax combined with second-line salvage immunochemotherapy and achieved CR/PR. Of note, twelve patients were treated with chimeric antigen receptor T-cell (CAR-T) therapy, and 58.3% obtained CR. At a median follow-up of 17.0 months, a majority of CAR-T patients (66.7%) maintained durable responses, while three patients died of disease progression or infectious complications.

### Immunosuppressive microenvironment in DHL/THL

To elaborate on the role of immune cells in DHL/THL, the tumor immunophenotyping (TIP) method was used to reveal the recruitment activity score of immune cells using RNA sequencing data from 27 patients, including 16 DHL/THL patients and 11 DHL-BCL6 patients. Although no significant differences in immune cell subsets were observed between groups, potentially attributable to limited cohort size, a tendency for increased infiltration of CD8 + T cells and CD4 + T cells was noted in DHL-BCL6 (Fig. S6a). Furthermore, compared to non-DHL controls, the DHL-BCL6 tumor microenvironment showed reduced immune cell infiltration, although no significant differences were observed in the lymphoma microenvironment (LME) subtypes (Fig. S7). Concordant with the prior study [12, 13], the depleted lymphoma microenvironment (LME-DP) subtype seemed to be more in patients with DHL/THL (36.4% in DHL-BCL6, 69.2% in DHL, and 66.7% in THL), which was characterized by a minimal presence of immune cells, as well as higher proportions of proliferating tumor cells and presented the worst prognosis (Fig. S6b-c). Gene set enrichment analysis (GSEA), as well as gene ontology (GO) pathway analysis, further revealed that immune-system-related pathways and cytokine signaling pathways were upregulated in DHL-BCL6, while the cell cycle pathway was enriched in DHL/THL (Fig. S6d-e).

## Discussion

High-grade B-cell lymphomas with MYC and BCL2 and/or BCL6 rearrangements represent aggressive entities. Significant differences in clinicopathologic and genetic features exist among this cohort. According to the 2022 WHO classification, DHL-BCL6 is no longer classified as DHL/THL and is categorized as DLBCL-NOS [14]. Thus, we collected the clinical and pathological data of 192 patients from 20 centers in China within the cooperative network of Multicenter Hematology-Oncology Programs Evaluation System (M-HOPES) to discern the disparities between DHL-BCL6 and DHL/THL, providing clinical and molecular evidence for different subgroups. DHL and THL shared overlapping clinicopathological profiles, while DHL-BCL6 was different. Most of the DHL/THL samples belonged to the GCB subtype, in contrast to the various COO subtypes in DHL-BCL6. In terms of prognosis, DHL/THL was associated with shorter PFS and OS than DHL-BCL6. These findings were consistent with results from another recently reported study in DHL/THL [15]. Multivariable analysis confirmed Ann Arbor stage and elevated LDH as independent determinants of inferior survival in the DHL/THL cohort. In contrast, LDH and COO subtypes emerged as prognostic biomarkers for DHL-BCL6 patients, potentially facilitating risk-adapted therapeutic intensification.

Emerging studies have delineated the genomic landscape of DHL/THL patients, revealing distinct mutational signatures in this high-risk population [12, 16, 17]. Importantly, our investigations further uncovered significant interpatient heterogeneity in genetic alterations. DHL/THL had comparable genetic characteristics, with the majority of genetic lesions being alterations associated with epigenetic regulation, such as *EZH2* and *CREBBP*. *EZH2* and *CREBBP*regulate histone modification, including methylation and acetylation, the loss of which leads to altered B-cell differentiation and proliferation [18, 19]. *EZH2* and *CREBBP*alterations can also cooperate with BCL2 overexpression to promote lymphomagenesis [20, 21]. Data from a phase I trial supported the therapeutic potential of multi-targeted agent combination in R/R DHL/THL, including venetoclax (BCL2 inhibitor), ibrutinib (B-cell receptor signaling inhibitor), obinutuzumab (anti-CD20 monoclonal antibody), and lenalidomide (immunomodulatory agent), especially in DHL [22]. In consistent with these observations, 4 R/R DHL/THL patients in our cohort responded well to a venetoclax-based regimen (venetoclax, chidamide combined with second-line salvage immunochemotherapy). Besides, a higher proportion of *TNFRSF14*mutations was more prevalent in THL patients, which inhibits Fas-induced apoptosis and is associated with immune escape [23]. As for the genetic landscape of DHL-BCL6, the genetic lesions were alterations associated with immune evasion and cell cycle, like *CD70* and *BTG2*. *CD70* suppresses T-cell-dependent cytotoxicity and indicates inferior outcomes in DHL-BCL6 [24]. *BTG2* regulates cell cycle arrest, with loss-of-function promoting malignant transformation [25]. Compared to DHL/THL, DHL-BCL6 possesses more complex biological properties, including various COO and molecular subtypes. Lenalidomide is an immunomodulatory agent with multiple potential mechanisms for anti-tumor activity [26]. It is worth mentioning that lenalidomide can safely be added to the R-DA-EDOCH regimen in aggressive B-cell lymphoma patients. Preliminary data showed that 9 (75.0%) of 12 enrolled patients achieved disease remission, and the OS was 93% with a median follow-up of 10.7 months [27], which supported the introduction of immunomodulatory agents into the treatment of DHL-BCL6. Meanwhile, DHL/THL was predominantly enriched in the LME-DP category, while the tumor microenvironment in DHL-BCL6 showed a trend of elevated CD4 + and CD8 + T cell infiltration. Moreover, pathway enrichment analysis of DHL-BCL6 revealed statistical enrichment of immune-related signaling pathways, including B-cell receptor (BCR) signaling and type I interferon-mediated signaling pathways. Collectively, these results suggest that DHL-BCL6 may be a more suitable candidate for microenvironment-mediated immunotherapy. Under the 2022 classification system, categorizing DHL-BCL6 as non-DHL, comparative analysis revealed distinct clinicopathological and molecular profiles in DHL-BCL6 versus non-DHL, despite comparable survival outcomes, suggesting that its unique biological behavior warrants further investigation. The distinct molecular heterogeneity between DHL-BCL6 and DHL/THL or non-DHL patients underscores the rationale for subtype-directed clinical trials and supports translating molecular profiling into precision oncology strategies, enabling biomarker-guided therapeutic optimization.

Given their resistance to rituximab-containing regimens, such as R-CHOP [28], DHL/THL require more effective immunochemotherapeutic approaches to improve clinical outcomes. A multicenter study evaluated the efficacy of induction therapy in 311 newly diagnosed DHL patients, of which 100 (32.2%) received R-CHOP, 65 (20.9%) received R-Hyper-CVAD, 64 (20.6%) received R-DA-EPOCH, and 42 (13.5%) received R-CODOX-M/IVAC. This research revealed that intensive regimens were associated with a significant reduction in progression risk and improvement in PFS [29]. In our study, patients who had clinical responses to first-line immunochemotherapy showed a remarkable improvement in survival. Consistent with previous reports (Table S5), we demonstrated that the R-DA-EDOCH regimen was associated with improved survival outcomes, which supported R-DA-EDOCH as a preferred first-line treatment option for patients with DHL and THL. In DHL-BCL6 patients, no significant survival difference was observed between R-DA-EDOCH and R-CHOP therapies, further highlighting the distinct clinical characteristics of DHL-BCL6, suggesting that treatment intensity requirements may differ from those of DHL/THL.

ASCT is a potentially curative strategy for patients with high-risk lymphomas. In DHL/THL, consolidative ASCT remains a matter of debate, and there is no strong evidence to indicate a beneficial impact on outcomes due to the limited sample size. Within the aforementioned study, a trend of improvement in survival is observed with first-line consolidation using ASCT [29]. Our cohort suggested ASCT may provide durable disease control in patients achieving CR or PR after induction therapy. Significantly superior survival was observed in ASCT recipients, with a 3-year PFS and 3-year OS rates of 84.8% and 90.9%, respectively. Despite minor age differences, no other differences in baseline characteristics existed between ASCT and non-ASCT patients. These findings suggest consolidative ASCT may be a potentially curative option for selected responders.

For patients with R/R DHL/THL, most patients develop resistance to chemotherapy and eventually die of rapid disease progression. CAR-T cell therapy emerges as an effective treatment modality for R/R DHL/THL, though longer follow-up is still required. In light of the poor prognosis, advances in therapeutics are urgently needed for R/R DHL/THL. Molecular profiling revealed that R/R DHL/THL patients exhibited a higher frequency of *TP53* mutations than those with sustained remission. The *TP53*gene plays a pivotal role in DNA repair, programmed cell death, cell cycle arrest, and genomic stability [30]. The clinical effectiveness of decitabine has been reported among DLBCL patients with *TP53*mutations, suggesting potential applicability [31]. Moreover, the selective exportin-1 (XPO-1) inhibitor selinexor was reported to induce upregulation of TP53 signaling in the treatment of DHL/THL [32]. Novel combinations incorporating targeted agents in R/R DHL/THL settings deserve more investigations in future studies.

Moreover, our study possesses acknowledged constraints, stemming from its retrospective design and the intrinsic limitations of an observational investigation. Selection bias could arise from the reliance on data sourced from a specific population. Besides, an inherent limitation is that FISH findings are probe-design-sensitive. Specifically, certain BCL6 FISH probes can misinterpret a single MYC rearrangement as dual oncogenic events by detecting both BCL6 promoter and MYC-BCL6 enhancer rearrangements, thereby inflating triple-hit lymphoma rates [33–36]. Multicenter prospective trials employing probes with different coverage are therefore warranted to validate preliminary proof-of-principle findings.

## Conclusion

Our cohort study of DHL/THL in China affirmed the highly aggressive nature and therapeutic resistance in this rare setting. The WHO 2022 classification redefined DHL-BCL6 as DLBCL-NOS, which was confirmed by our results that DHL/THL show a higher frequency of *EZH2* mutation and possess a worse prognosis than DHL-BCL6. However, intensified treatment strategies and consolidative ASCT significantly improve survival in DHL/THL. In contrast, DHL-BCL6 patients display diverse molecular subtypes and comparable survival outcomes to those with DLBCL-NOS, responding well to both R-CHOP and R-DA-EDOCH regimens. Moreover, our results emphasized the importance of molecular profiling for prognosis and personalized therapy, paving the way for more precise and effective interventions in this challenging disease.

## Materials and methods

### Patients

This study enrolled 192 newly diagnosed DHL/THL patients between January 2014 and June 2024, with follow-up censored on October 1, 2024. The cases were sourced from the 20 M-HOPES consortium centers. Histological diagnoses were rendered per the WHO 2022 classification criteria [37], including DHL (*n* = 71) with MYC and BCL2 translocations and THL (*n* = 41) with concomitant MYC, BCL2, and BCL6 translocations. DHL-BCL6 (*n*= 80) was also included for comparison. The non-DHL control cohort, excluding DHL-BCL6, comprised 955 patients derived from the LymphPlex algorithm framework. Clinical information, including gender, age, IPI score, Ann Arbor stage, ECOG performance status, LDH level, extranodal sites, immunochemotherapy regimens, therapeutic efficacy, and survival time, was collected. Positron emission tomography-computed tomography (PET-CT) served as the modality for treatment response evaluation [38]. IHC was examined to classify all cases as non-GCB or GCB phenotypes using the Hans algorithm [39]. Double-expressor lymphomas were defined as BCL-2 expression ≥ 50% and MYC expression ≥ 40% of the tumor cells by IHC [40]. Sustained remission was defined as the absence of disease progression from the end of treatment until the last follow-up.

### FISH

To detect key genetic alterations in lymphomagenesis, interphase FISH was carried out on 3-μm tissue paraffin sections. Gene rearrangements involving MYC, BCL2, and BCL6 were screened using a break-apart probe assay. Specific translocations, including MYC/IGH and BCL2/IGH, were subsequently confirmed with dual-color, dual-fusion probes (All probes were obtained from Guangzhou LBP, China; Codes: F.01150–01, F.01063–01, and F.01066–01). The threshold for defining chromosomal translocations was set as the mean value of split signals in control samples plus three standard deviations. The cutoff for positivity with the break-apart probes was established at 10%. Samples demonstrating ≥ 3 fusion signals in ≥ 10% of cells received amplification-positive classification [41].

### DNA sequencing

All DNA sequencing data were collected from Ruijin hospital. Genomic DNA extraction employed the QIAamp DNA Microkit (Qiagen, Duesseldorf, Germany) for frozen tumors or the GeneRead DNA FFPE Kit (Qiagen) for formalin-fixed and paraffin-embedded (FFPE) tissues. Matched blood samples underwent Sanger sequencing of tumor-driver mutations to exclude germline variants. Six high-quality frozen/FFPE tumor specimens were subjected to whole-exome sequencing (WES), with exome capture using the SeqCap EZ Human Exome Kit v3.0 on Righton's HiSeq 4000 platform (Shanghai). Eight patients underwent whole genome sequencing (WGS) in frozen tumor tissue, with genomic DNA concentrations measured using Thermo Fisher technology and then clipped into fragments of about 300 bp using the Covaris DNA clipping system. After terminal repair and 3 'terminal adenylation, the Illumina PE adapter is attached to the DNA fragment to generate an index library. Library integrity was confirmed via the Agilent 2100 Bioanalyzer, with subsequent sequencing conducted on Wuxi NextCODE's Illumina HiSeq platform. Targeted sequencing covering 55 lymphomagenesis-related genes was conducted on 88 FFPE tumor specimens, with DNA integrity verified through agarose gel electrophoresis. Primer design employed Primer 5.0 software. The amplicon library was derived from the multiplex PCR amplification system of Shanghai Riton Biopharmaceutical, and deep sequencing utilized the HiSeq 4000 platform. The RefSeq database (Human Reference 96 Genome version hg19) served as the reference genome. A custom pipeline developed with this software detected single nucleotide variants (SNVs) and identified insertions/deletions.

### Molecular classification

Incorporating mutational profiles from 35 genes with FISH-detected BCL2, BCL6, and MYC rearrangements, we designed a simplified model with 38 genes designed for lymphoma typing (LymphPlex algorithm). According to the LymphPlex algorithm, DLBCL were identified with seven genetic subtypes: EZB-like (including EZB-like MYC- and EZB-like MYC +), MCD-like, BN2-like, N1-like, ST2-like, TP53^Mut^, or NOS [11]. The LymphPlex algorithm is accessible online at: https://kylinmu.shinyapps.io/LymphPlexR/. Compared to the LymphGen algorithm [42], the LymphPlex algorithm achieved 95% accuracy with higher unique subtype classification rates (81.4% vs 67.6%), simplified workflow, and broader subtype coverage (Fig. S8a-b). Besides, we also applied the novel DLBclass tool for molecular subtyping, which shared certain defining molecular features with the LymphGen subtypes [43]. Similar to those reported in the literature, 50% of C1 DLBCLs were classified as BN2-like; 100% of C2 tumors were designated as *TP53*^MUT^; 64% of C3 DLBCLs were defined as EZB-like; 25% of C4 tumors were identified as ST2-like; and 20% of C5 DLBCLs were classified as MCD (Fig. S8c).

### RNA sequencing and bioinformatic analysis

All RNA sequencing data were collected from Ruijin hospital. Total RNA extraction from 27 frozen tumor specimens employed Trizol and RNeasy Mini Kits (Qiagen), stratifying patients into 13 DHL, 3 THL, and 11 DHL-BCL6 subtypes. RNA quantification utilized Nanodrop, while integrity assessment leveraged the Agilent 2100 Bioanalyzer. Burrows-Wheeler Aligner (v0.7.13-r1126) aligned read pairs to RefSeq hg19. Bioinformatic analyses were conducted using R 3.6.1, which implemented the "sva" method for batch effect correction. Limma (v3.38.3) normalized raw reads, yielding differentially expressed genes (DEGs). Detailed processes for RNA sequencing were carried out as previously reported [44]. Tumor microenvironment was analyzed using the TIP method (http://biocc.hrbmu.edu.cn/TIP) [45]. RNA-seq-identified patients underwent stratification into four LME categories per established criteria: germinal center-like (LME-GC), mesenchymal (LME-ME), inflammatory (LME-IN), and LME-DP [13].

### Statistical analysis

Statistical analyses were conducted using SPSS 26.0 (IBM, Chicago, IL). Categorical and continuous variables were summarized as frequency (percentage) and median (range), respectively. Pearson's χ2-test or Fisher's exact tests ascertained clinical and genetic characteristics. PFS encompassed the interval from diagnosis to disease progression, relapse, or death. OS spanned the period from diagnosis to all-cause mortality. Kaplan–Meier estimates with log-rank testing were analyzed to examine survival outcomes, with censoring curves plotted at 60 months due to insufficient numbers of at-risk patients. Cox proportional hazards regression was used to model associations between predictors and recurrence/death risks. Variables demonstrating statistical significance in univariate analysis, along with IPI-associated clinical factors, were incorporated into the multivariate analysis model. Mann–Whitney U tests were used to compare intergroup immunity scores and normalized gene expression. The statistical significance threshold was *p* < 0.05.

## Supplementary Information

Supplementary Material 1.

## Data Availability

Genomic and transcriptomic datasets have been deposited on China National Center for Bioinformation, under project accession number PRJCA042679 (https://ngdc.cncb.ac.cn/bioproject/). De-identified participant data underlying study findings are available upon reasonable request via zhao.weili@yahoo.com.
