# Supplementary material for: Clinicopathological characteristics, genetic aberrations, and optimized treatment strategies in double-hit and triple-hit lymphoma: a multi-center cohort study
Source: Mol Biomed. 2025 Dec 9;6:137. doi: 10.1186/s43556-025-00346-8 (PMC12690028; doi:10.1186/s43556-025-00346-8)
Supplement: Supplementary file 1 — Supplementary Material 1. [file 43556_2025_346_MOESM1_ESM.docx]

**Supplementary Material for**

**Clinicopathological characteristics, genetic aberrations, and optimized treatment strategies in double-hit and triple-hit lymphoma: a multi-center cohort study**

Yi-Ge Shen, Meng-Meng Ji, Qing Shi, Xiao-Lei Wei, Lei Fan, Ting-Bo Liu, Yao Liu, Li-Hua Dong, Ai-Bin Liang, Liang Huang, Hui Zhou, Hong-Hui Huang, Shen-Miao Yang, Xiao-Bo Wang, Yu-Yang Tian, Zun-Min Zhu, Ou Bai, Fei Li, Wen-Yu Shi, Bin Xu, Xin Wang, Ke-Qian Shi, Wei Tang, Hong-Mei Yi, Si-Yuan Chen, Zhong Zheng, Shu Cheng, Peng-Peng Xu, Wei-Li Zhao^, Li Wang^

^ Correspondence author. Email: [wl11194@rjh.com.cn](mailto:wl11194@rjh.com,cn) and [zhao.weili@yahoo.com](mailto:zhao.weili@yahoo.com)

**Supplementary Tables**

| Characteristic | non-DHL  (n=955) n (%) | DHL-BCL6 (n=80) n (%) | P value |
| --- | --- | --- | --- |
| Age, y |  |  |  |
| Median (range) | 57 (16-92) | 58 (26-81) |  |
| ≤60 | 552 (57.8) | 44 (55.0) | 0.6263 |
| ＞60 | 403 (42.2) | 36 (45.0) |  |
| Gender |  |  |  |
| Male | 514 (53.8) | 47 (58.8) | 0.3954 |
| Female | 441 (46.2) | 33 (41.2) |  |
| IPI risk group |  |  |  |
| 0-2 | 668 (69.9) | 47 (58.8) | 0.0374 |
| 3-5 | 287 (30.1) | 33 (41.2) |  |
| ECOG |  |  |  |
| <2 | 860 (90.1) | 70 (87.5) | 0.4676 |
| ≥2 | 95 (9.9) | 10 (12.5) |  |
| Ann Arbor stage |  |  |  |
| I-II | 527 (55.2) | 28 (35.0) | 0.0005 |
| III-IV | 428 (44.8) | 52 (65.0) |  |
| Extranodal sites |  |  |  |
| <2 | 706 (73.9) | 50 (62.5) | 0.0269 |
| ≥2 | 249 (26.1) | 30 (37.5) |  |
| LDH |  |  |  |
| ≤normal | 513 (53.7) | 35 (43.8) | 0.0862 |
| >normal | 442 (46.3) | 45 (56.2) |  |
| Cell of origin |  |  |  |
| GCB | 136/366 (37.2) | 38 (47.5) | 0.0858 |
| Non-GCB or ABC | 230/366 (62.8) | 42 (52.5) |  |
| BCL2/MYC DE |  |  |  |
| With | 317/905 (35.0) | 42 (52.5) | 0.0019 |
| Without | 588/905 (65.0) | 38 (47.5) |  |

**Table S1. Comparative analysis of clinicopathologic features between non-DHL and DHL-BCL6 Patients**

Abbreviations: DHL, double hit lymphoma; DHL-BCL6, DLBCL with MYC and BCL6 rearrangements; IPI, International Prognostic Index; ECOG, eastern cooperative oncology group; LDH, lactate dehydrogenase; GCB, germinal center B-cell; ABC, activated B-cell like; DE, MYC/BCL2 protein co-expression.

| Category | Variables | Progression-free survival | |  | |  | Overall survival | |  | |
| --- | --- | --- | --- | --- | --- | --- | --- | --- | --- | --- |
|  |  | Univariate |  | Multivariate |  |  | Univariate |  | Multivariate |  |
|  |  | HR (95% CI) | P value | HR (95% CI) | P value |  | HR (95% CI) | P value | HR (95% CI) | P value |
| Age, y | ≤60 | 1 |  | 1 |  |  | 1 |  | 1 |  |
|  | >60 | 1.162 (0.530-2.548) | 0.7084 | 0.927 (0.391-2.200) | 0.8635 |  | 2.397 (0.700-8.205) | 0.1637 | 1.843 (0.486-6.983) | 0.3684 |
| Gender | Female | 1 |  | - |  |  | 1 |  | - |  |
|  | Male | 1.164 (0.513-2.639) | 0.7161 | - | - |  | 0.704 (0.209-2.375) | 0.5713 | - | - |
| IPI risk group | 0-2 | 1 |  | - |  |  | 1 |  | - |  |
|  | 3-5 | 1.766 (0.804-3.880) | 0.1566 | - | - |  | 1.401 (0.422-4.647) | 0.5817 | - | - |
| ECOG | <2 | 1 |  | 1 |  |  | 1 |  | 1 |  |
|  | ≥2 | 3.008 (1.198-7.548) | 0.0190 | 2.506 (0.895-7.014) | 0.0802 |  | 4.325 (1.262-14.826) | 0.0198 | 2.976 (0.772-11.471) | 0.1132 |
| Ann Arbor stage | I-II | 1 |  | 1 |  |  | 1 |  | 1 |  |
|  | III-IV | 2.672 (1.000-7.140) | 0.0499 | 2.031 (0.701-5.886) | 0.1921 |  | 3.141 (0.667-14.789) | 0.1477 | 2.438 (0.462-12.859) | 0.2934 |
| Extranodal sites | <2 | 1 |  | 1 |  |  | 1 |  | 1 |  |
|  | ≥2 | 0.739 (0.319-1.715) | 0.4818 | 0.561 (0.219-1.435) | 0.2277 |  | 0.582 (0.154-2.197) | 0.4244 | 0.331 (0.078-1.414) | 0.1357 |
| Serum LDH | ≤normal | 1 |  | 1 |  |  | 1 |  | 1 |  |
|  | >normal | 4.459 (1.665-11.941) | 0.0029 | 3.185 (1.128-8.989) | 0.0287 |  | 2.692 (0.710-10.211) | 0.1455 | 2.542 (0.613-10.540) | 0.1986 |
| Cell of origin (Hans) | GCB | 1 |  | 1 |  |  | 1 |  | - |  |
|  | Non-GCB | 3.493 (1.444-8.453) | 0.0055 | 2.738 (1.099-6.822) | 0.0306 |  | 2.947 (0.775-11.212) | 0.1128 | - | - |
| BCL2/MYC DE | Without | 1 |  | - |  |  | 1 |  | - |  |
|  | With | 0.895 (0.408-1.962) | 0.7811 | - | - |  | 1.034 (0.315-3.396) | 0.9558 | - | - |

**Supplementary Table 2. Univariate analysis and multivariate analysis of prognostic factors in DHL-BCL6 patients**

Abbreviations: HR, hazard ratio; CI, confidence interval; IPI, International Prognostic Index; ECOG, eastern cooperative oncology group; LDH, lactate dehydrogenase; GCB, germinal center B-cell; DE, MYC/BCL2 protein co-expression; DHL-BCL6, DLBCL with MYC and BCL6 rearrangements.

| Characteristic | Sustained remission  DHL/THL patients  (n=57) | R/R DHL/THL (n=55) | P value |
| --- | --- | --- | --- |
| Age, y |  |  |  |
| Median (range) | 53 (22-79) | 55 (31-81) |  |
| ≤60 | 38 (66.7) | 35 (63.6) | 0.7365 |
| ＞60 | 19 (33.3) | 20 (36.4) |  |
| Gender |  |  |  |
| Female | 27 (47.4) | 22 (40.0) | 0.4320 |
| Male | 30 (52.6) | 33 (60.0) |  |
| IPI risk group |  |  |  |
| 0-2 | 37 (64.9) | 22 (40.0) | 0.0083 |
| 3-5 | 20 (35.1) | 33 (60.0) |  |
| ECOG |  |  |  |
| <2 | 49 (86.0) | 46 (83.6) | 0.7313 |
| ≥2 | 8 (14.0) | 9 (16.4) |  |
| Ann Arbor stage |  |  |  |
| I-II | 20 (35.1) | 11 (20.0) | 0.0774 |
| III-IV | 37 (64.9) | 44 (80.0) |  |
| Extranodal sites |  |  |  |
| <2 | 33 (57.9) | 29 (52.7) | 0.5824 |
| ≥2 | 24 (42.1) | 26 (47.3) |  |
| LDH |  |  |  |
| ≤normal | 20 (35.1) | 13 (23.6) | 0.1839 |
| ＞normal | 37 (64.9) | 42 (76.4) |  |
| Cell of origin (Hans) |  |  |  |
| GCB | 50 (87.7) | 43 (78.2) | 0.1788 |
| Non-GCB | 7 (12.3) | 12 (21.8) |  |
| BCL2/MYC DE |  |  |  |
| With | 43 (75.4) | 45 (81.8) | 0.4108 |
| Without | 14 (24.6) | 10 (18.2) |  |
| First-line therapy |  |  |  |
| R-DA-EDOCH | 32 (56.1) | 18 (32.8) | 0.0060 |
| R-CHOP/R-CHOP like | 17 (29.8) | 33 (60.0) |  |
| R-CHOP+X | 7 (12.3) | 2 (3.6) |  |
| IR2/ZR2 | 1 (1.8) | 2 (3.6) |  |

**Supplementary Table 3. Clinicopathologic features of sustained remission and R/R DHL/THL patients**

Abbreviations: DHL, double hit lymphoma; THL, triple hit lymphoma; IPI, International Prognostic Index; ECOG, eastern cooperative oncology group; LDH, lactate dehydrogenase; GCB, germinal center B-cell; DE, MYC/BCL2 protein co-expression; R-CHOP, rituximab with cyclophosphamide, doxorubicin, vincristine, and prednisone; R-CHOP+X, R-CHOP+novel targeted agents; R-DA-EDOCH, rituximab, etoposide, vincristine, doxorubicin, cyclophosphamide, and dexamethasone; IR2, ibrutinib, rituximab, and lenalidomide; ZR2, zanubrutinib, rituximab, and lenalidomide.

|  | 3-year PFS | |  | 3-year OS | |  |
| --- | --- | --- | --- | --- | --- | --- |
|  | ASCT | non-ASCT | P value | ASCT | non-ASCT | P value |
| DHL-BCL6 | 94.1% | 68.1% | 0.1309 | 100.0% | 90.1% | 0.1618 |
| DHL | 87.3% | 38.0% | 0.0033 | 92.9% | 61.5% | 0.0594 |
| THL | 80.2% | 40.2% | 0.1414 | 85.7% | 35.6% | 0.0976 |

**Supplementary Table 4. Outcomes of patients received autologous stem cell transplant according to predefined subgroups**

Abbreviations: PFS, progression free survival; OS, overall survival; ASCT, autologous stem cell transplantation; DHL-BCL6, DLBCL with MYC and BCL6 rearrangements; DHL, double hit lymphoma; THL, triple hit lymphoma.

| Patient (including DHL/THL and DHL-BCL6) | Treatment | Conclusion | References |
| --- | --- | --- | --- |
| 311 | R-CHOP (n = 100) vs intense regimens (R-DA-EPOCH (n = 65), R-Hyper CVAD (n = 64), R-CODOX-M/IVAC (n = 42)) | Intensive regimens were associated with significantly improved PFS, but not OS. | Petrich et al. (2014) |
| 159 | R-CHOP (n = 35) vs intense regimens (DA-EPOCH-R (n = 81), R-Hyper CVAD (n = 32), R-CODOX-M/IVAC (n = 11)) | 3-year RFS was inferior in patients who received R-CHOP compared with intensive therapy | Landsburg et al. (2017) |
| 129 | R-CHOP (n = 57) vs R-DA-EPOCH (n = 28) vs  R-Hyper CVAD (n = 34) | R-EPOCH was associated with longer EFS compared to R-CHOP. | Oki et al. (2014) |
| 60 | R-CHOP (n = 19) vs R-Hyper CVAD (n = 28) | No survival benefit was showed from DA-EPOCH-R comparing to R-CHOP | Li et al. (2012) |
| 32 | R-CHOP (n = 5) vs R-CODOX-M/IVAC (n = 25) | Patients with double-hit lymphoma treated with CODOX-M/IVAC+R followed by hematopoietic cell transplantation can achieve durable remissions. | Sun et al. (2015) |
| 192 | R-CHOP (n = 83) vs R-DA-EDOCH (n = 87) | Results supported use of more intensive regimens like R-DA-EPOCH over R-CHOP when treating DHL patients. | Our study |

**Supplementary Table 5. Arrangement of literature**

**
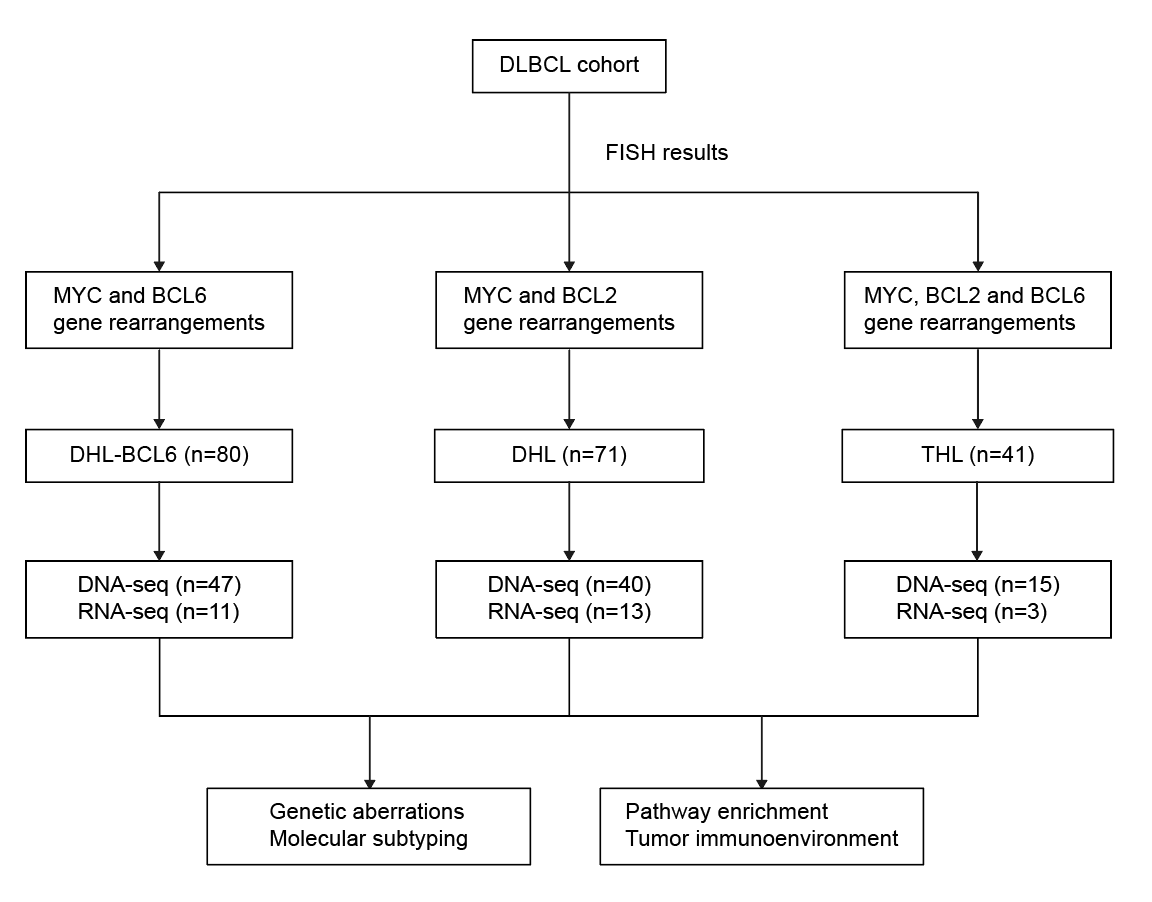
Supplementary Figures**

**Supplementary Figure 1. Flowchart of cohort selection and exploratory aspects**


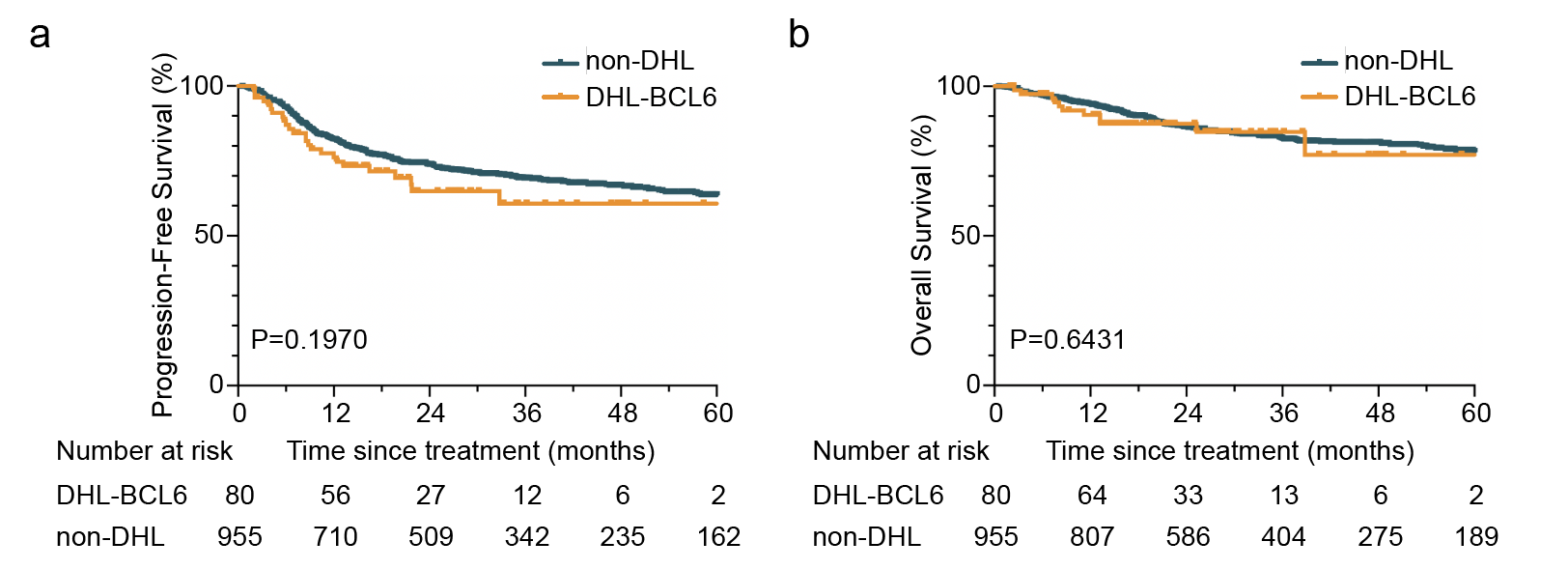
**Supplementary Figure 2. Comparative analysis of survival outcomes between non-DHL and DHL-BCL6 Patients**

Kaplan-Meier curves of PFS (a) and OS (b) among non-DHL patients (n=955) and DHL-BCL6 patients (n=80).

Abbreviations: PFS, progression free survival; OS, overall survival; DHL, double hit lymphoma; DHL-BCL6, DLBCL with MYC and BCL6 rearrangements.

**
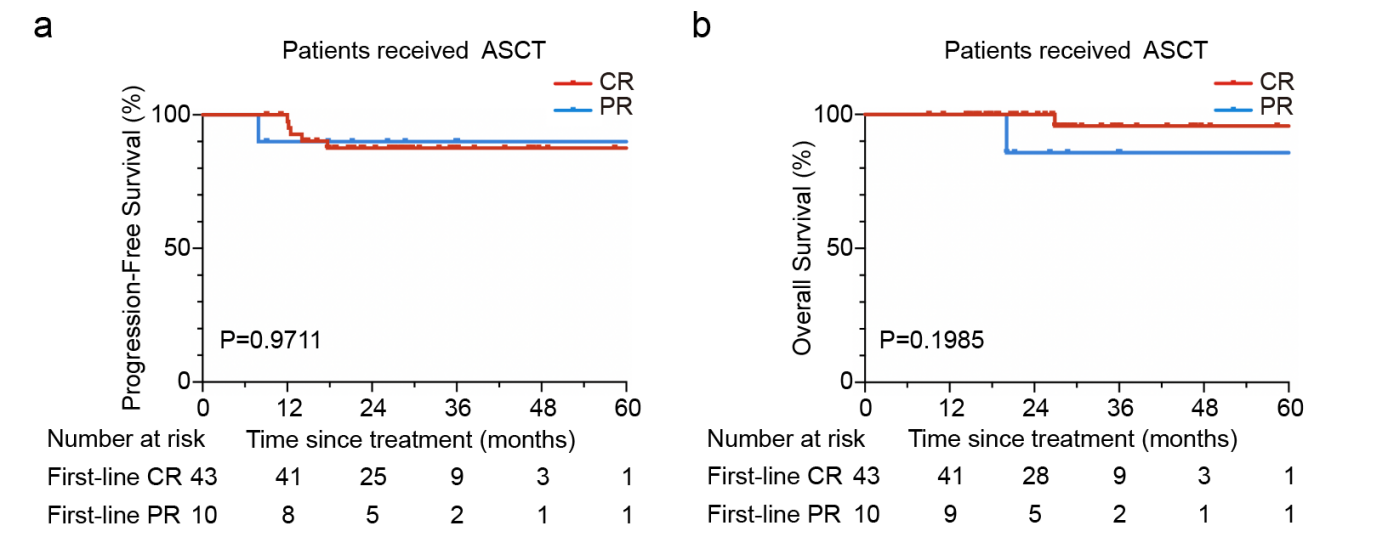
Supplementary Figure 3. Survival outcomes of patients received autologous stem cell transplant according to treatment responses**

Kaplan-Meier curves of PFS (a) and OS (b) in total patients achieved complete remission (CR) (n=43) and in those achieved partial remission (PR) (n=10) before ASCT.

Abbreviations: PFS, progression free survival; OS, overall survival; ASCT, autologous stem cell transplantation; CR, complete remission; PR, partial remission.

**
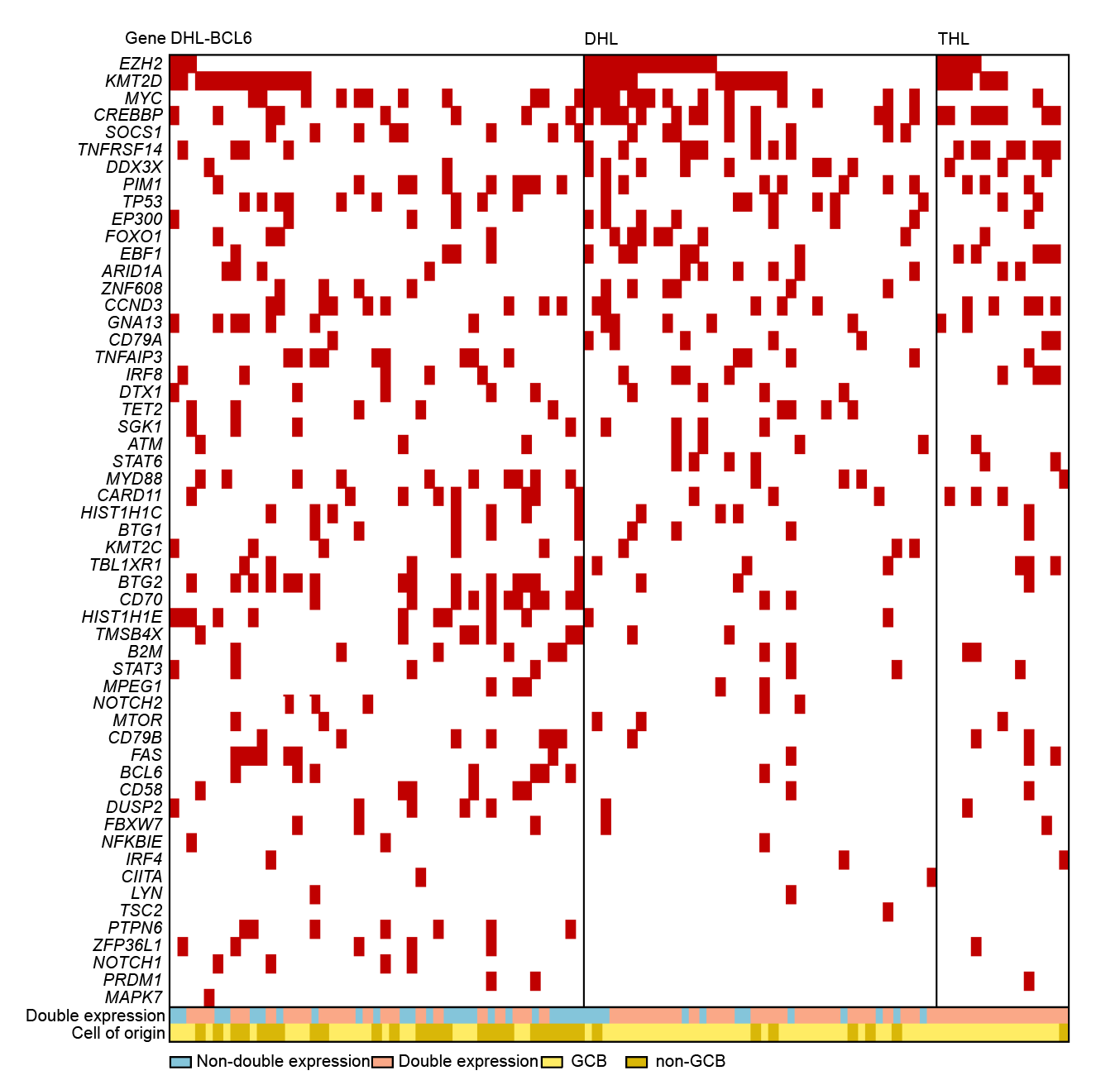
**

**Supplementary Figure 4. Mutation profile of DHL-BCL6, DHL and THL patients**

**
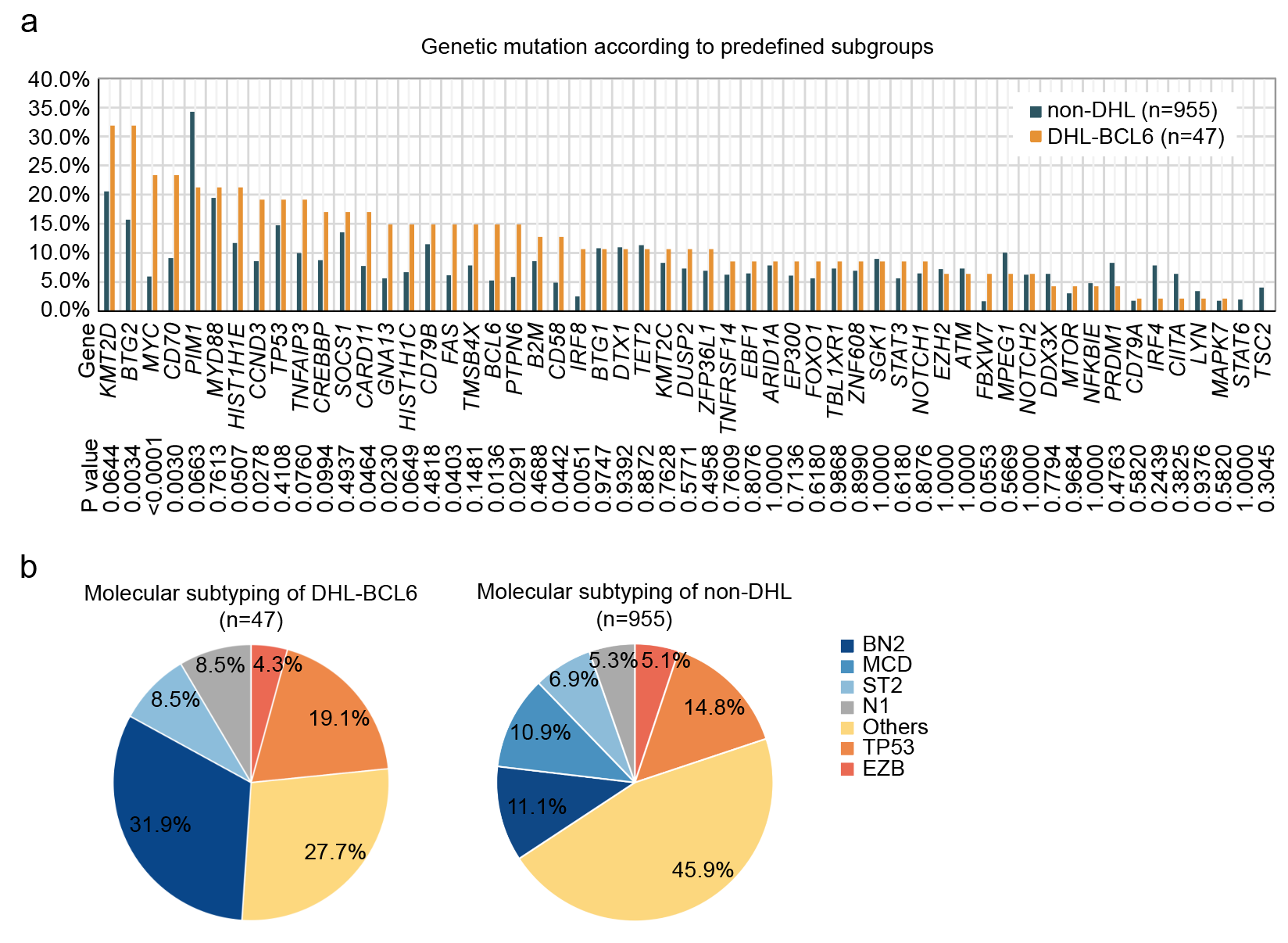
Supplementary Figure 5. Comparative analysis of genetic mutation and molecular subtyping between non-DHL and DHL-BCL6 Patients**

1. Prevalence of genetic mutations in patients with non-DHL (n=955) and DHL-BCL6 (n=47). Lower graph indicates P values comparing different prevalence in two groups. (b) Prevalence of DLBCL subtypes classified by LymphPlex in non-DHL (n=955) and DHL-BCL6 (n=47).


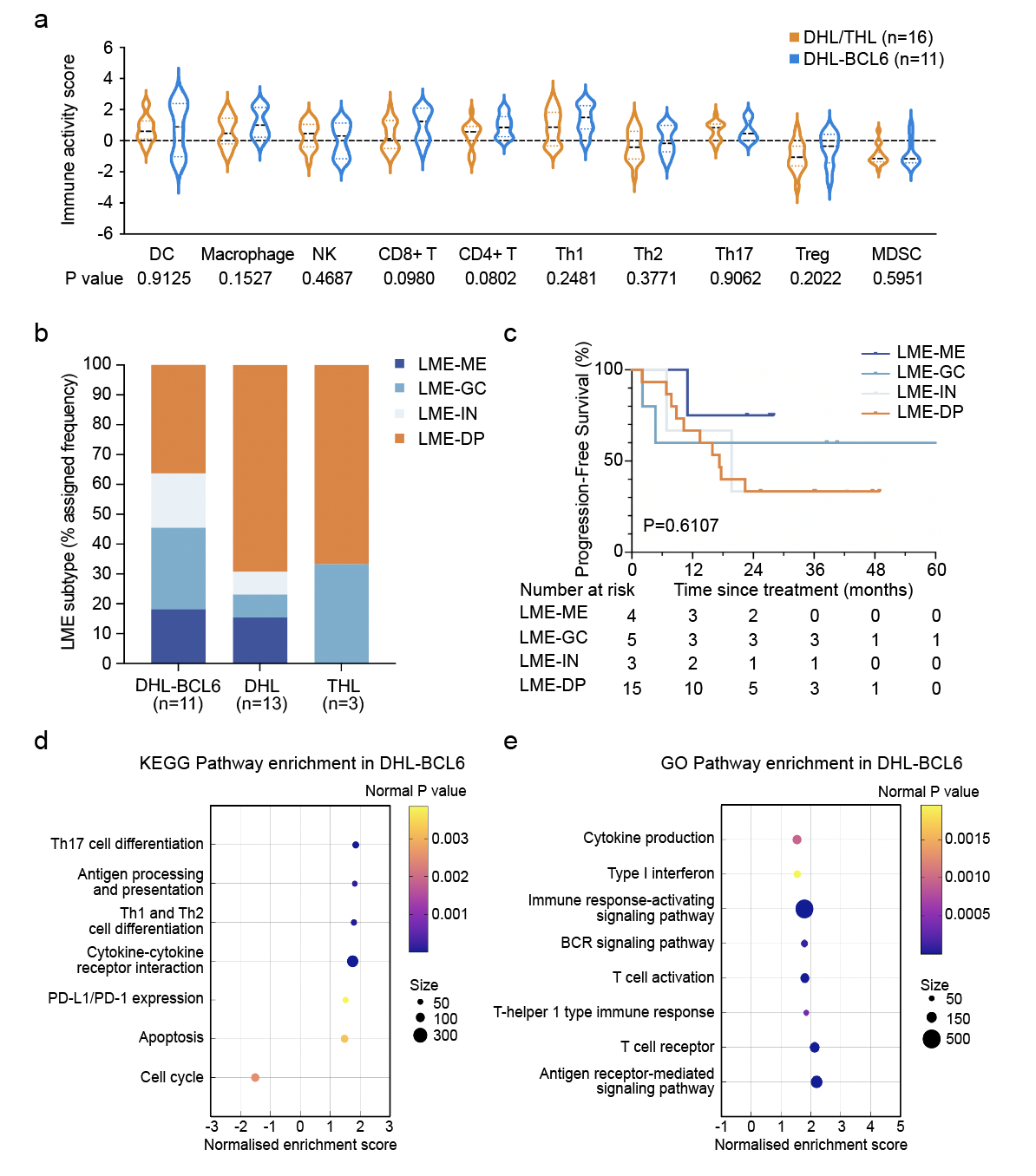

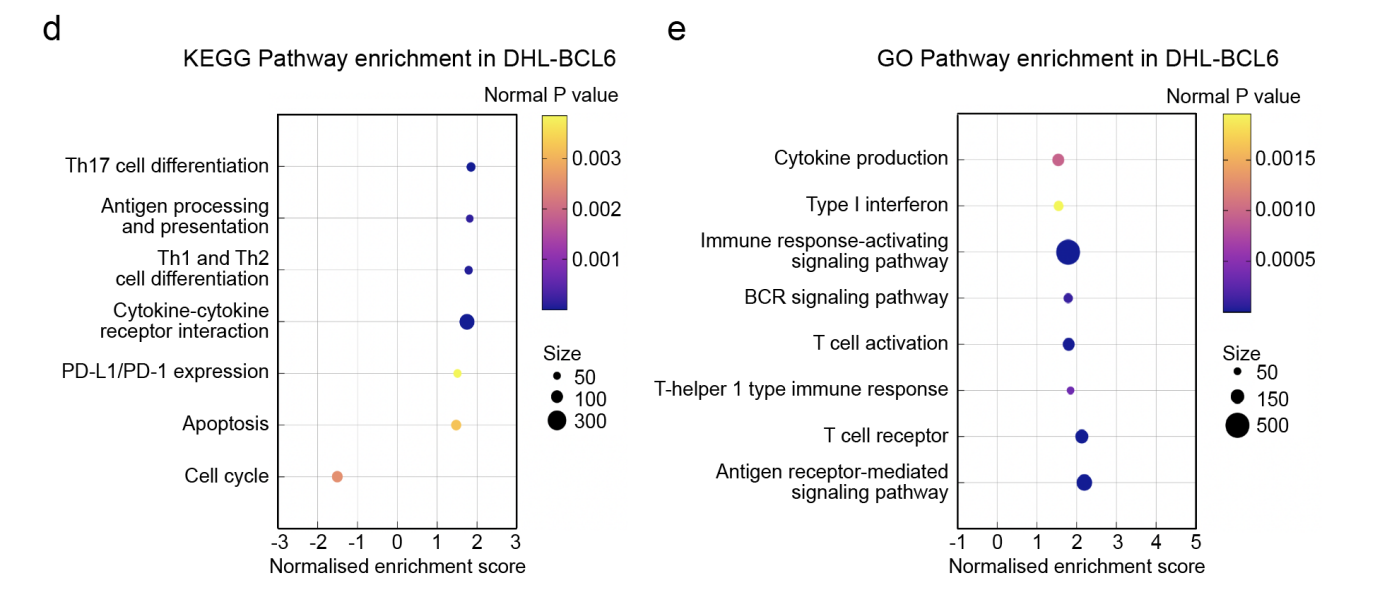


**Supplementary Figure 6. Immune microenvironment analysis between DHL/THL and DHL-BCL6 patients**

1. Immunity activity scores of immune cells in DHL/THL (n=16) and DHL-BCL6 (n=11) patients. The lower graph indicates p-values comparing different scores in two groups.
2. Prevalence of DLBCL subtypes classified by LME categories.
3. Kaplan-Meier analysis of progression-free survival according to LME categories.

(d-e) Comparison of gene set enrichment analysis in patients with DHL/THL (n=16) and DHL-BCL6 (n=11). Gene set enrichment analysis showed the upregulated pathways in DHL-BCL6 group.

Abbreviations: LME, lymphoma microenvironment; DHL-BCL6, DLBCL with MYC and BCL6 rearrangements; DHL, double hit lymphoma; THL, triple hit lymphoma.

**
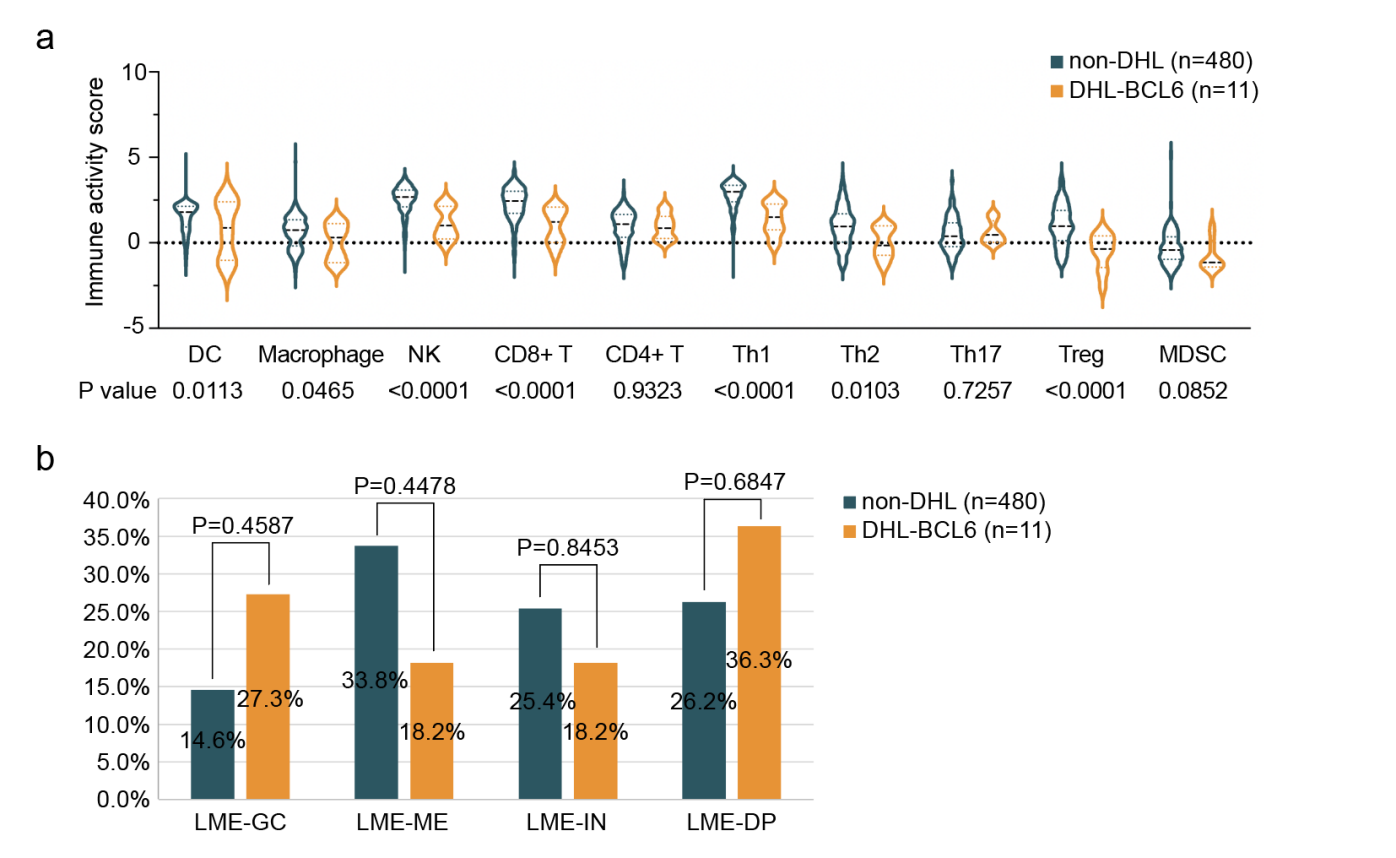
**

**Supplementary Figure 7. Immune microenvironment analysis between non-DHL and DHL-BCL6 patients**

1. Immunity activity scores of immune cells in non-DHL (n=480) and DHL-BCL6 (n=11) patients. The lower graph indicates p-values comparing different scores in two groups.
2. Prevalence of DLBCL subtypes classified by LME categories.

Abbreviations: DHL, double hit lymphoma; DHL-BCL6, DLBCL with MYC and BCL6 rearrangements; LME, lymphoma microenvironment.

**
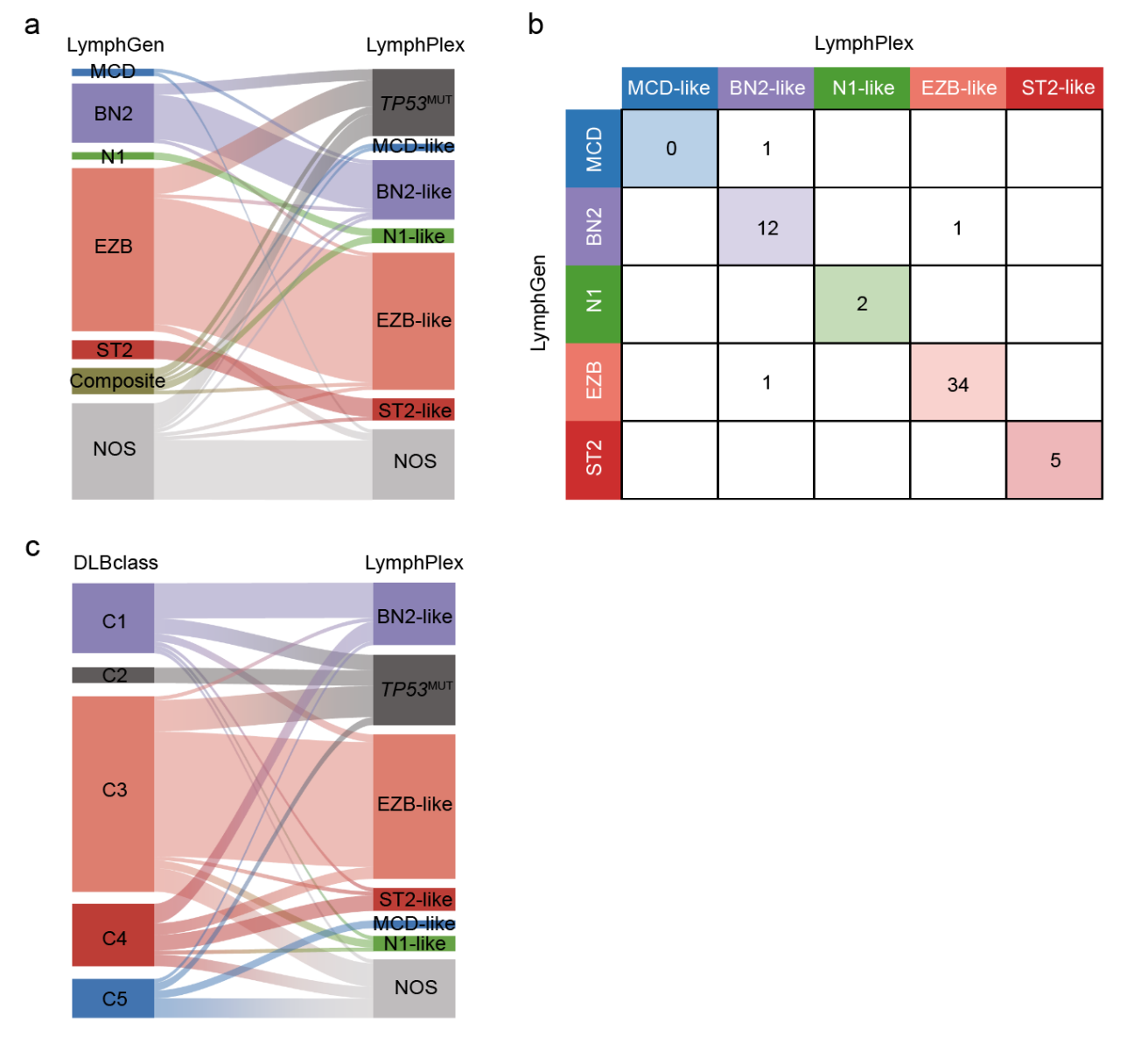
Supplementary Figure 8. Validation of the genetic subtyping using LymphPlex algorithm and LymphGen algorithm**

1. Sankey plots showing the results of the LymphPlex algorithm and the LymphGen algorithm in 102 patients with DNA sequencing.
2. Confusion matrices indicate sample distribution across subtypes predicted by LymphPlex algorithm versus LymphGen algorithm.
3. Sankey plots showing the results of the LymphPlex algorithm and the DLBclass algorithm in 102 patients with DNA sequencing.
